# Supplementary material for: MarShie: a clearing protocol for 3D analysis of single cells throughout the bone marrow at subcellular resolution
Source: Nat Commun. 2024 Feb 26;15:1764. doi: 10.1038/s41467-024-45827-6 (PMC10897183; doi:10.1038/s41467-024-45827-6)
Supplement: Supplementary file 1 — Supplementary Information [file 41467_2024_45827_MOESM1_ESM.pdf]

Supplementary Information

MarShie: a clearing protocol for 3D analysis of single cells throughout the bone marrow at subcellular resolution

Till Fabian Mertens *[1,2], Alina Tabea Liebheit *[1,2,3], Johanna Ehl [1,2], Ralf Köhler [2], Asylkhan Rakhymzhan [1,4], Andrew Woehler [5,6], Lukas Katthän [7], Gernot Ebel [7], Wjatscheslaw Liublin [4], Ana Kasapi [1,8], Antigoni Triantafyllopoulou [1,8], Tim Julius Schulz [9,10], Raluca Aura Niesner #[4,11], Anja Erika Hauser #[1,2]

Affiliations

1) Department of Rheumatology and Clinical Immunology, Charité - Universitätsmedizin Berlin, corporate member of Freie Universität Berlin and Humboldt-Universität zu Berlin, 10117 Berlin, Germany
2) Immune Dynamics, Deutsches Rheuma-Forschungszentrum (DRFZ), a Leibniz Institute, Chariteplatz 1, 10117 Berlin, Germany
3) Institute of Chemistry and Biochemistry, Department of Biology, Chemistry and Pharmacy, Freie Universität Berlin, Germany
4) Biophysical Analytics, Deutsches Rheuma-Forschungszentrum (DRFZ), a Leibniz Institute, Charitéplatz 1, 10117 Berlin, Germany
5) Berlin Institute for Medical Systems Biology, Max Delbrück Center for Molecular Medicine, 10115 Berlin, Germany
6) Current address: Janelia Research Campus, Howard Hughes Medical Institute, Ashburn, VA 20147, USA
7) Miltenyi Biotec B.V. and Co. Bertha-von-Suttner-Straße 5, 37085 Göttingen
8) Innate Immunity in Rheumatic Diseases, Deutsches Rheuma-Forschungszentrum (DRFZ), a Leibniz Institute, Charitéplatz 1, 10117 Berlin, Germany
9) Department of Adipocyte Development and Nutrition, German Institute of Human Nutrition (DIfE) Potsdam-Rehbruecke, 14558 Nuthetal, Germany
10) German Center for Diabetes Research (DZD), 85764 Munich-Neuherberg, Germany
11) Dynamic and Functional in vivo Imaging, Veterinary Medicine, Freie Universität Berlin, Berlin, Germany

*The authors contributed equally to this work

#The authors jointly supervised this work

Corresponding author Anja E. Hauser, anja.hauser-hankeln@charite.de

Supplementary Figures

Supplementary Figure 1

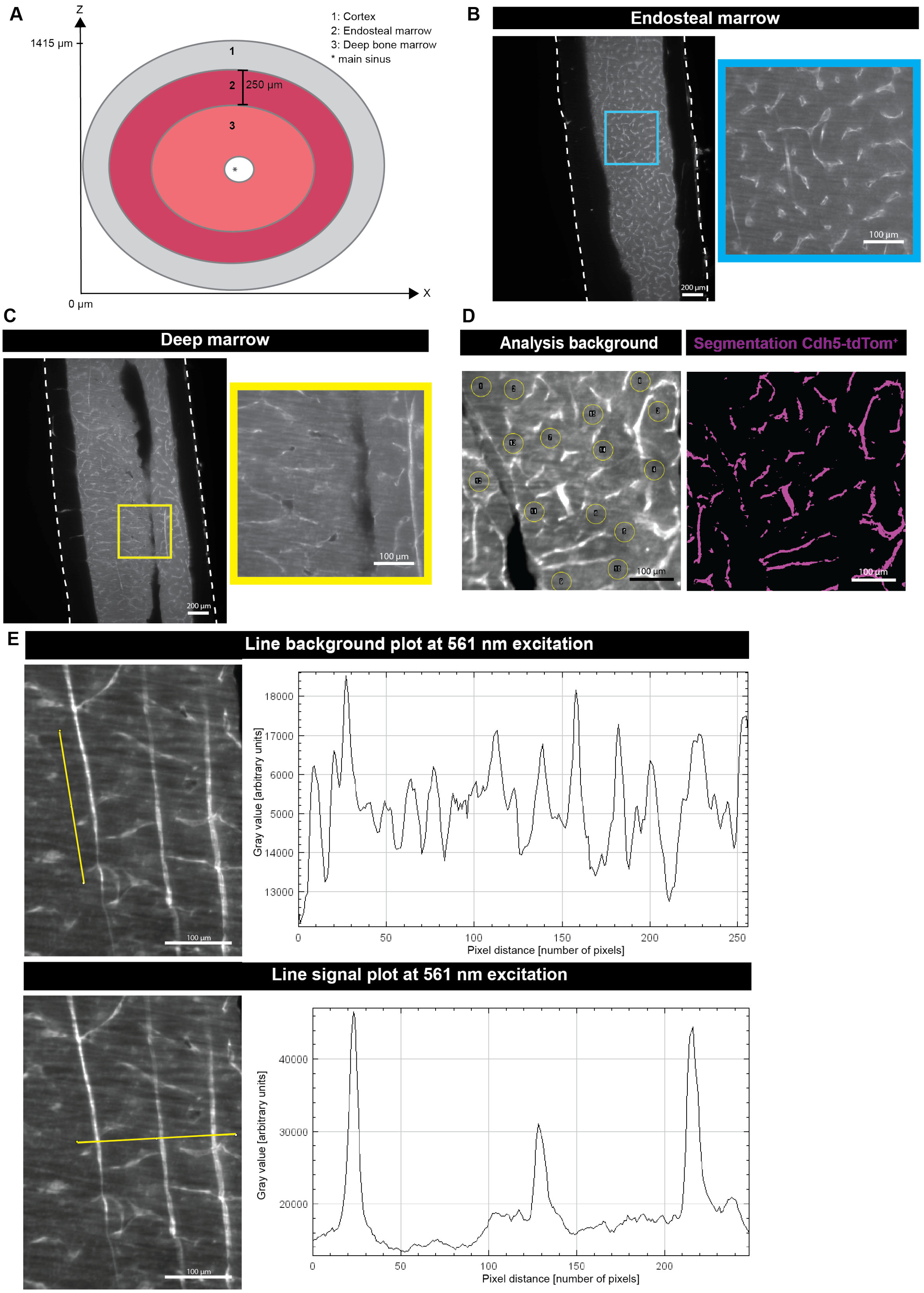

Supplementary Figure 1. BM regions and strategy for signal quantification.

a, Schematic for the compartmentalization of the transverse BM sections into concentric endosteal and deep marrow regions, according to their location within the BM. **b,** Examples for the position of ROIs chosen from endosteal (blue square) regions. Raw data for Cdh5-tdTom fluorescence; outer cortical borders are marked with white dashed lines. **c,** Examples for the position of ROIs chosen from deep BM (yellow square) regions. Raw data for Cdh5-tdTom fluorescence; outer cortical borders are marked with white dashed lines. **d**, Examples (yellow circles in left image) for the selection of ROIs (n=15 for three z- levels each) classified as background. Raw data for Cdh5-tdTom fluorescence. Right image: Example for LABKIT assisted segmentation of Cdh5-tdTom$^+$ endothelial cells. **e**, Upper left panel: Example of contour (yellow) chosen to generate a line plot for local background intensities at 561 nm excutation. Right plot: pixel intensities over distance. Lower left panel: example of contour (yellow) chosen to generate a line plot for quantification of the Cdh5-tdTomato signal. Right plot: signal intensities over distance.

Supplementary Figure 2

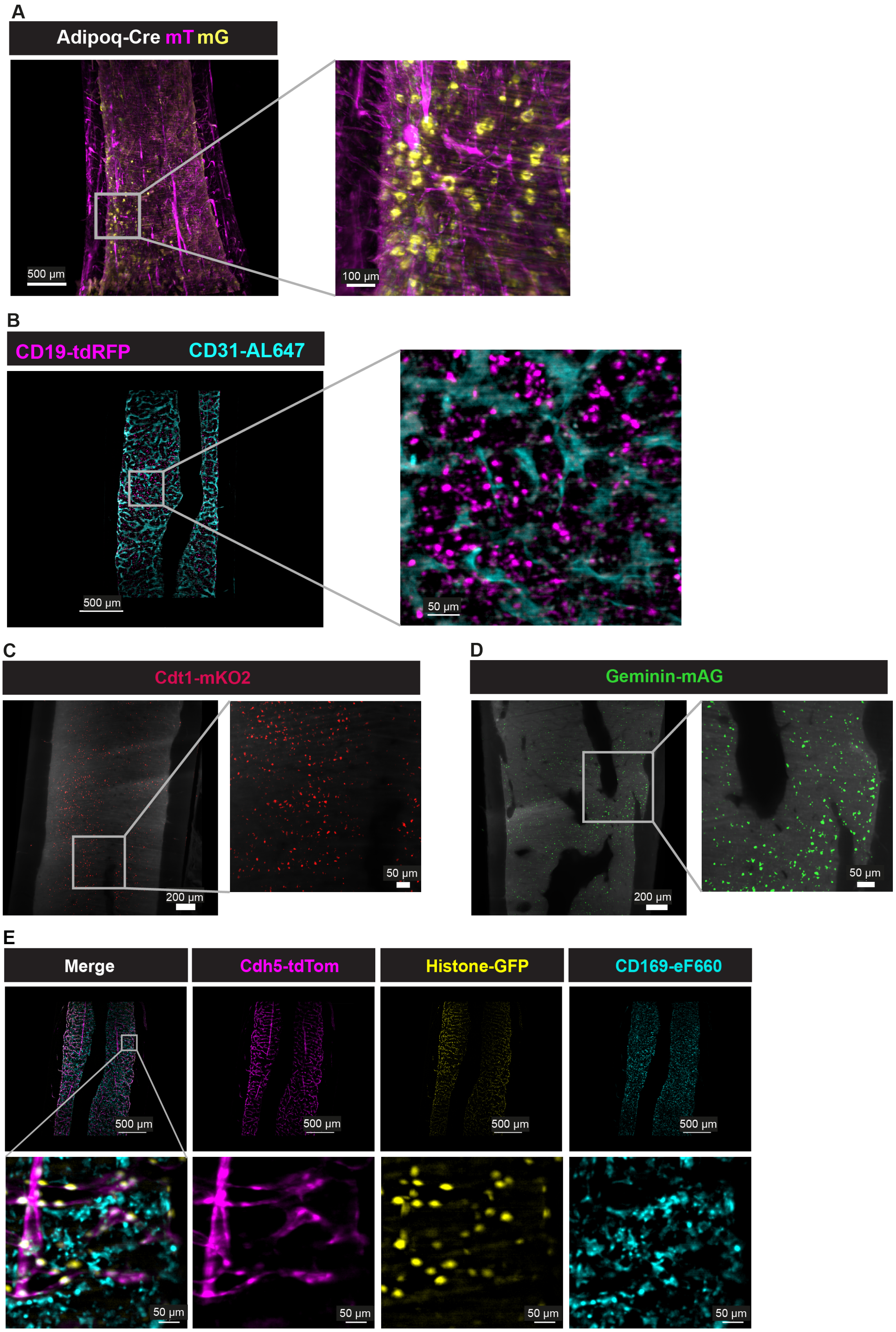

Supplementary Figure 2. MarShie is compatible with various fluorescent proteins and with staining using fluorophore-labeled antibodies.

a, Representative 3D reconstruction of a femur from an Adipoq-Cre-mTmG mouse, showing tissue structures in magenta (mT$^+$) and Adiponectin-Cre labeled, GFP$^+$ cells (mG$^+$) with an ovoid morphology in yellow. Some mG+ cells contain non-fluorescent vacuoles. In this young mouse, adipocytes are mainly present in the lower part of the femur. Image representative of 4 animals analyzed. **b,** Representative xy-orthogonal slices (30 µm thick) of deep BM regions from a CD19-tdRFP mouse injected with an anti-CD31 antibody conjugated to AexaFluor(AL)647. CD19-tdTom signal in magenta (8 µm background subtracted), CD31-AL647 signal in cyan (25 µm background subtracted). Zoom-in demonstrates the distribution of tdRFP$^+$ cells in the parenchyma. Three female mice were used in 3 independent experiments. **c,** Representative image of deep BM from Fucci-expressing hCdt1-mKO2 (639) mouse, n=4 from 3 different experiments. **d,** Representative image of deep BM from a hGem-mAG (Fucci 474) mouse, n=4 from 3 different experiments. **e,** Representative xy-orthogonal slices (30 µm thick) of deep BM regions from a Cdh5-tdTom/Histone-GFP mouse injected with an anti-CD169 antibody conjugated to eF660. Cdh5-tdTom signal in magenta (25 µm background subtracted), endothelial histone GFP signal in yellow (5 µm background subtracted), CD169-eF660 signal in cyan (12 µm background subtracted). Three female mice were used, each in a separate experiment.

Supplementary Figure 3

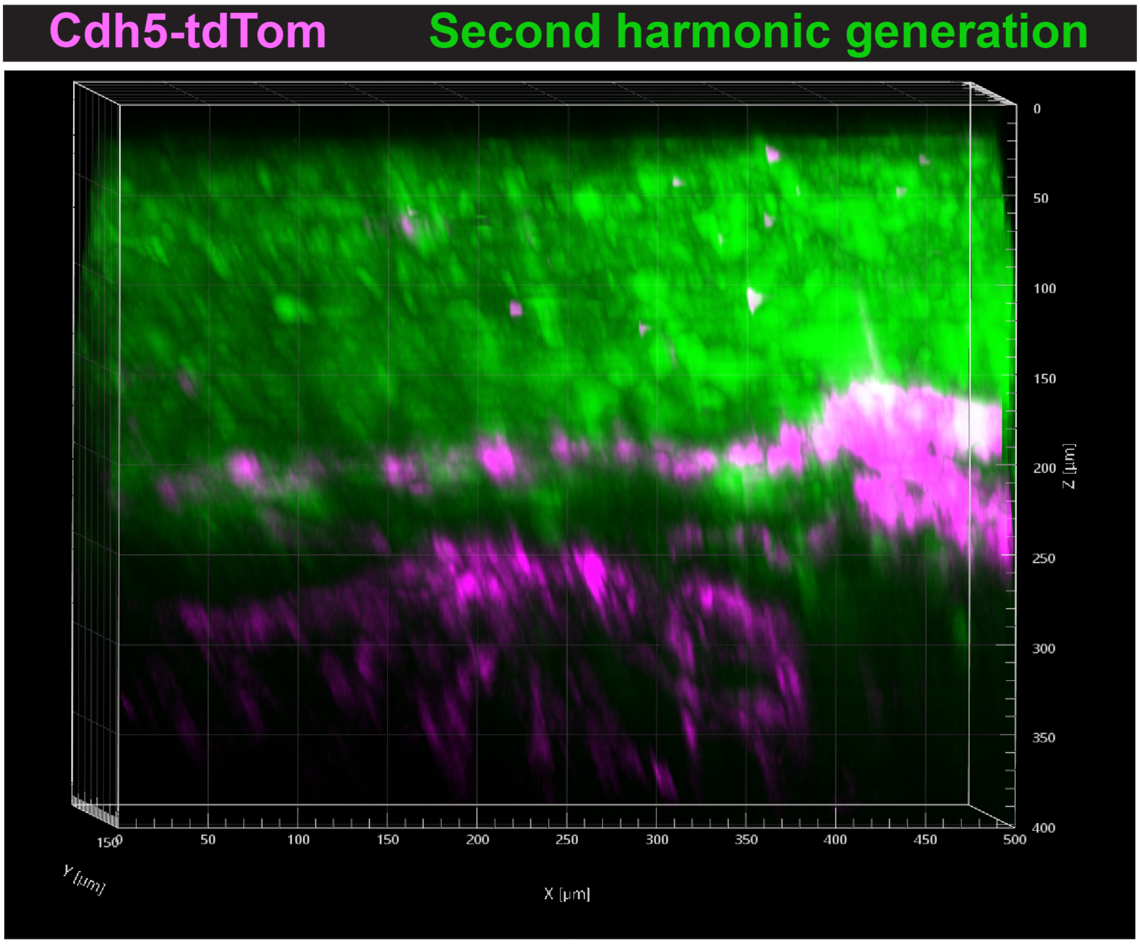

Supplementary Figure 3. Two-photon imaging of a MarShie-cleared femur derived from a Cdh5-TdTom mouse demonstrates preservation of cortical collagen structures.

3D reconstruction of endothelia expressing tdTom (magenta) and collagen fibers, visualized by SHG (green). The achieved imaging depth in the tdTom channel amounts to ~400 µm, covering ~230 µm bone cortex (SHG) and ~170 µm BM, as shown by the scale.

Supplementary Figure 4

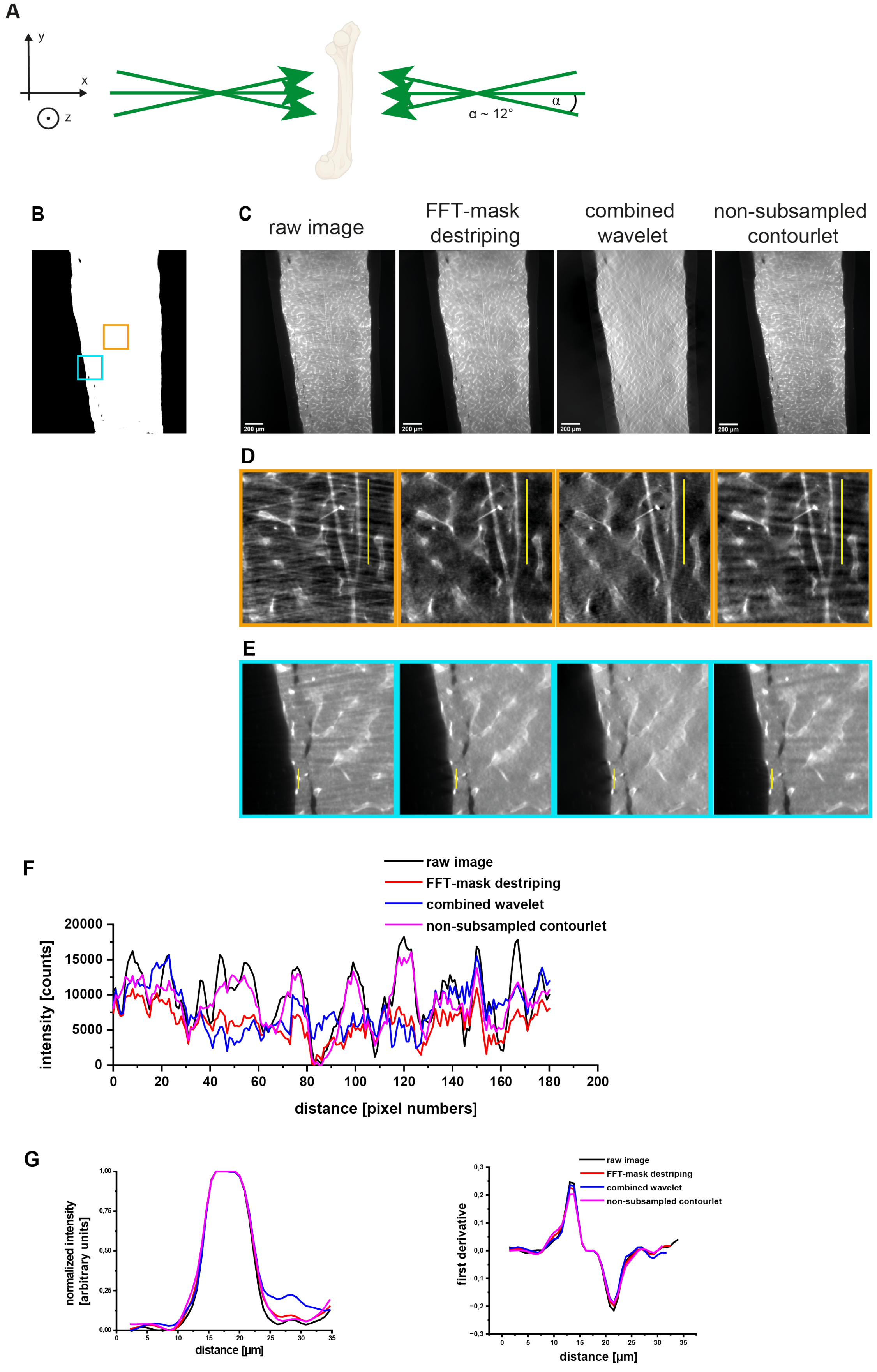

Supplementary Figure 4. Comparison of de-striping methods based on depicted example of the BM in a Cdh5-tdTomato mouse.

a, Schematic illumination orientation of the six beam paths in the light sheet microscope. The laser configuration dictates stripe artifact alignment. Illustration created with BioRender.com. **b,** Mask image depicting the areas chosen to represent endost (cyan frame) and deep BM (orange frame), **c,** Images before and after applying various de-striping algorithms. From left to right: raw image, directional FFT de-striping approach, combined wavelet transform approach [31] extended by directional mask removal in the FFT space, and non-subsampled contourlet transform approach [32]. **d**, Zoom-ins of the areas depicted within the orange frame in panel b. Yellow line marks the area analyzed in panel (f). **e,** Zoom-ins of the areas depicted within the cyan frame in panel b. Yellow line marks the area analyzed in panel (g). **f,** Line profiles from the images in d, demonstrating stripe removal by the directional FFT de-striping and the combined wavelet transform approaches, but not by non-subsampled contourlet transform. Source data are provided as a Source Data file. **g,** Left graph: line profiles of a small structure in the endosteal area from the images in (e), and first derivatives of the line profiles (right graph) show that all approaches used for stripe artefact removal preserve spatial resolution. Scale bars are 200 µm. Source data are provided as a Source Data file.

Supplementary Figure 5

A

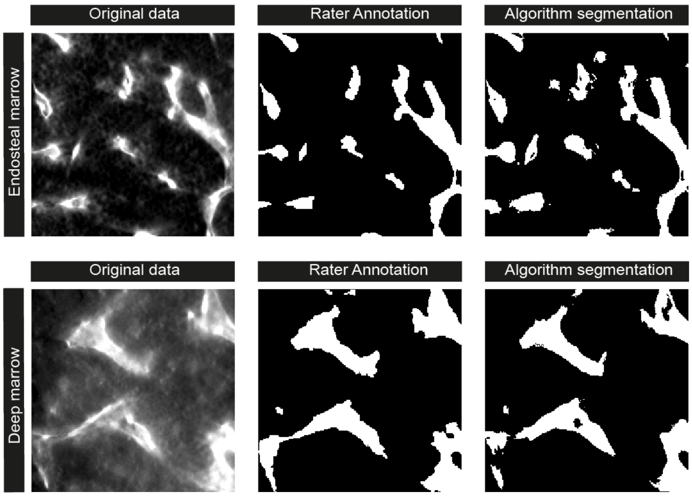

B

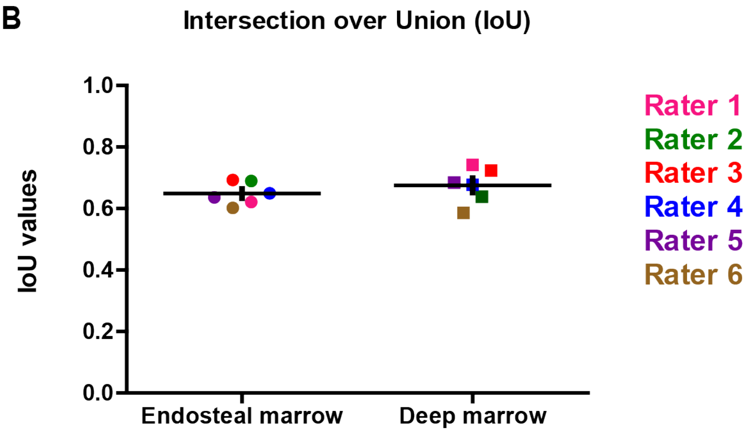

C

Interrater variability (IoU values)

	Rater 1	Rater 2	Rater 3	Rater 4	Rater 5	Rater 6
Rater 1	1	0,72	0,66	0,71	0,68	0,6
Rater 2	0,72	1	0,68	0,69	0,73	0,68
Rater 3	0,66	0,68	1	0,67	0,64	0,63
Rater 4	0,71	0,69	0,67	1	0,67	0,61
Rater 5	0,68	0,73	0,64	0,67	1	0,63
Rater 6	0,6	0,68	0,63	0,61	0,63	1

Supplementary Figure 5. Validation of the segmentation algorithm.

a, Examples of endosteal (upper panels) and deep BM regions (lower panels) used for image classification. Original data are shown on the left. An example of rater annotation are presented in the middle, segmentation by the LABKIT algorithm is presented in panels on the right. **b,** IoU analysis comparing the segmentation results of six trained raters to the performance of the LABKIT algorithm, in endosteal and deep BM regions (z-stacks consisting of n=10 consecutive levels). An identical segmentation would result in an IoU value of 1. Source data are provided as a Source Data file.**c,** Interrater variability values, determined by comparing the segmentation results between individual raters using IoU.

Supplementary Figure 6

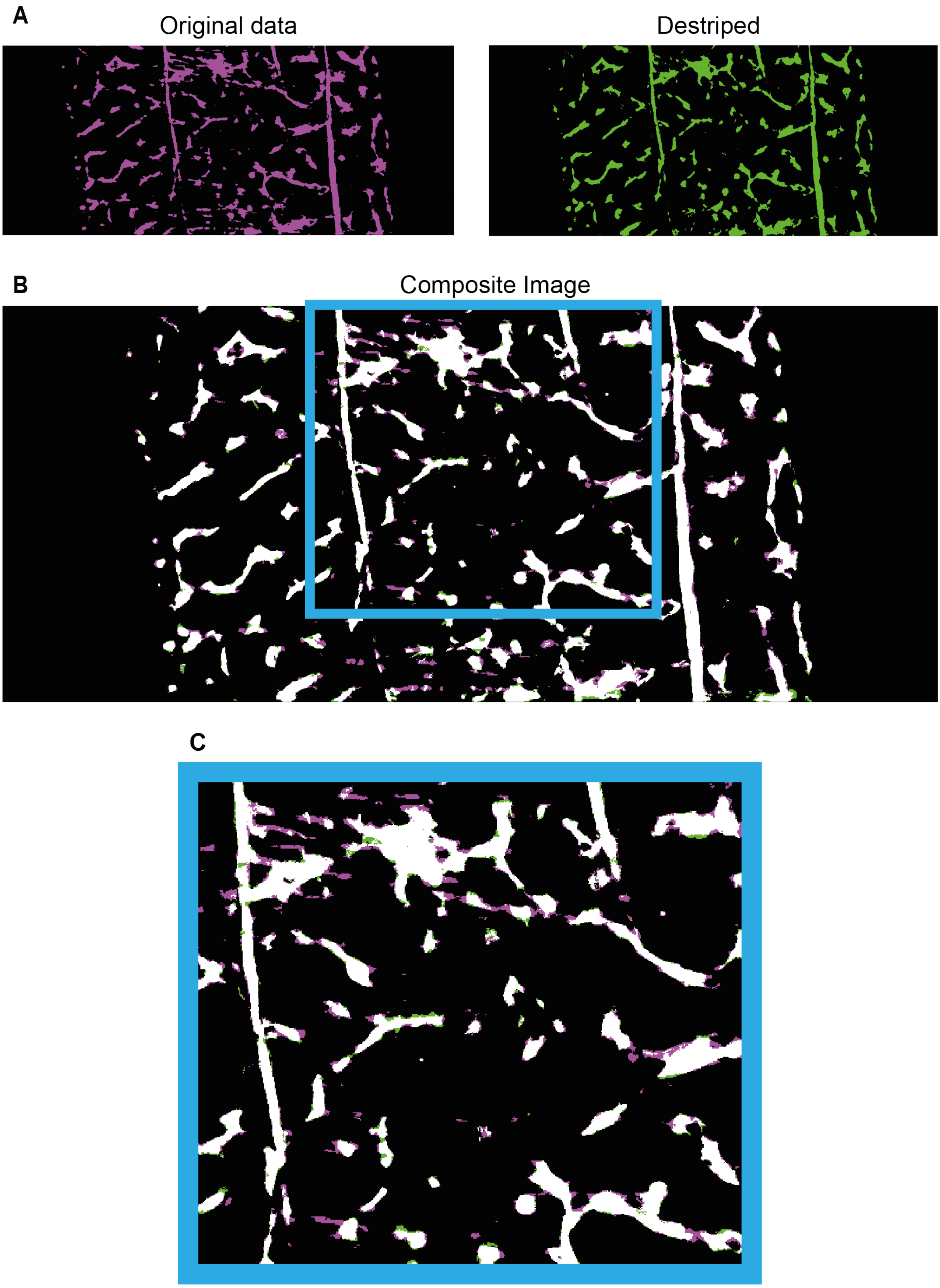

Supplementary Figure 6. De-striping removes artifacts that are otherwise classified as vessels.

a, Examples for segmented images using original image data (magenta) and destriped data (green). **b,** Overlay of both images highlights the signal removed by de-striping. Signals present in both images are shown in white, signals present only before de-striping are depicted in magenta. **c,** Magnification of the area within the blue frame in (b) highlights the striped signals which the algorithm recognizes as vascular structures.
