# Supplementary material for: MarShie: a clearing protocol for 3D analysis of single cells throughout the bone marrow at subcellular resolution
Source: Nat Commun. 2024 Feb 26;15:1764. doi: 10.1038/s41467-024-45827-6 (PMC10897183; doi:10.1038/s41467-024-45827-6)
Supplement: Supplementary file 3 — Description of Additional Supplementary Files [file 41467_2024_45827_MOESM3_ESM.pdf]

Description of Additional Supplementary Files

File name: Supplementary Software 1
Description: De-striping Algorithm.

This script suppresses stripes in light sheet fluorescence microscopy (LSFM) images based on a directional Gaussian two-dimensional fast Fourier transform (FFT) filter.
LSFM used: Ultramicroscope II (LaVision Biotec / Miltenyi, Germany)

Name: destripeLSFM_byDirectionalGaussianFFTfilter_stack.pyPython Environment used:

Python 3.9.7
numpy 1.20.3
scikit-image 0.18.3
scipy 1.7.1
matplotlib 3.4.3 # necessary for pylab

The microscope imposed parameters for the Ultramicroscope II contain:

angles = [100, 80, 90] # illumination angles
angular_diff = 0.048 # divergence of scattered light
line_width = 3.4 # standard deviation describing light sheet width in FFT

Parameters have to be adapted for other image data from different microscopic setups.

Script application

Open the script in your python environment matching at least the requirements mentioned above.
When running the script, it will ask the user where single images are located. After selection of desired folder containing all single images, sequential suppression of stripes in each image is performed. Resulting images are stored under the same location in a newly created folder called "destriped".

Script was applied on Win10, 4 Core @ 2.4 GHz, 8 GB RAM; Expected runtime per image ~ 45s.

File name: Supplementary Movie 1
Description: 3D rendering and orthogonal xy-slicing of a MarShie-cleared murine diaphyseal femur volume from a CD19-tdRFP mouse injected with an anti-CD31 antibody conjugated to AL-647 imaged by light sheet microscopy. CD19-tdTom signal in magenta (8 µm background subtracted), CD31-AL647 signal in cyan (25 µm background subtracted). Resolution 0.94 µm/pixel, z-step size 5 µm. Image volume: 2.1 mm x 2.4 mm x 1.32 mm.

File name: Supplementary Movie 2
Description: 3D rendering and orthogonal xy-slicing of a MarShie-cleared murine diaphyseal femur volume from a Cdh5-TdTom mouse injected with an anti-CD169 antibody conjugated to eFluor660. Cdh5-tdTom signal in magenta (25 µm background subtracted), endothelial histone-GFP in yellow (5 µm background subtracted), CD169-eF660 signal in cyan (12 µm background subtracted). Resolution 0.94 µm/pixel, z-step size 5 µm. Image volume: 2.1 mm x 2.4 mm x 1.42 mm.

File name: Supplementary Movie 3
Description: Two-photon imaging of a MarShie-cleared murine diaphyseal femur volume femur derived from a Cdh5-TdTom mouse demonstrates preservation of cortical collagen structures. Orthogonal xy-slicing of endothelia expressing tdTom (magenta) and collagen fibers, visualized by SHG (green). The achieved imaging depth in the tdTom channel amounts to ~400 µm, covering ~230 µm bone cortex (SHG) and ~170 µm BM, as shown by the scale.

File name: Supplementary Movie 4
Description: 3D rendering of the vascular network in an entire MarShie-cleared murine femur, imaged by light sheet microscopy. Cdh5-tdTom vessels in magenta form the vascular network throughout the whole femur. Resolution 2.41 µm/pixel, z-step size 5 µm. Image volume: 4.6 mm x 16.9 mm x 3.3 mm.

File name: Supplementary Movie 5
Description: Orthogonal xy-slicing of a MarShie-cleared murine diaphyseal femur volume from a Cdh5-tdTom-Histone-GFP mouse. Cdh5-tdTom+ vessels are shown in magenta, Histone-linked GFP+ of endothelial nuclei in white. Visualization shows the vascular network, which comprises transcortical, sinusoidal and arterial vessels as well as the central sinus. 30 µm xy-orthogonal slices, 0.94 µm/pixel, z-step size 3 µm, Image volume: 2 mm x 2.4 mm x 1.7 mm.

File name: Supplementary Movie 6
Description: 3D rendering and orthogonal xy-slicing of the segmented vessel network including endothelial nuclei of a MarShie-cleared murine femur diaphysis from a CX3CR1-GFP x Cdh5-tdTom-Histone-GFP mouse. 3D segmentation of Cdh5tdTom+ vessels (magenta) and histone-linked GFP+ endothelial nuclei (white) within the marrow, after excluding the surrounding cortex. Zoom-ins show segmented endothelial nuclei and vessels. 0.94 µm/pixel, z-step size 3 µm. Image volume: 2 mm x 0.5 mm x 2 mm.

File name: Supplementary Movie 7
Description: Orthogonal xy-slicing through a MarShie-cleared femoral diaphysis from a young (20 weeks old) mouse. Cdh5-tdTom+ vessels are shown in magenta, autofluorescence signal of marrow and cortical structures at 488 nm excitation is displayed in grey. Segmentation of the central sinus is marked by white lines. 0.94 µm/pixel, z-step size 3 µm. Image volume: 1.9 mm x 2 mm x 1.6 mm.

File name: Supplementary Movie 8
Description: Orthogonal xy-slicing through a MarShie-cleared femoral diaphysis from an old (86 weeks old) mouse. Cdh5-tdTom+ vessels are shown in magenta, autofluorescence signal of marrow and cortical structures at 488 nm excitation is displayed in grey. The segmentation of the diminished and displaced central sinus is marked by white lines. 0.94 µm/pixel, z-step size 3 µm. Image volume: 1.9 mm x 2 mm x 1.7 mm.

File name: Supplementary Movie 9
Description: 3D rendering and orthogonal xy-slicing of a cortical region highlighting the intracortical vessel network in an old mouse. Cdh5-tdTom+ vessels shown in magenta, autofluorescence signal of marrow and cortical structures at 488 nm excitation is displayed in grey. 0.94 µm/pixel, z-step size 3 µm. Image volume: 2 mm x 2 mm x 1.4 mm. At the time of sacrifice, the mouse was 86 weeks old.

File name: Supplementary Movie 10
Description: 3D rendering and orthogonal xy-slicing of segmented myeloid cells and the vascular network in a diaphyseal volume from a CX3CR1-GFP x Cdh5-tdTomato/histone-GFP mouse. 3D segmentation of CX3CR1-GFP+ myeloid cells in green and of Cdh5-tdTom+ vessels in magenta within the marrow, after excluding the surrounding cortex. Zoom-in shows morphology of myeloid cells and their position with respect to the vessels. 0.94 µm/pixel, z-step 3 µm. Image volume: 2 mm x 0.5 mm x 2 mm.

File name: Supplementary Movie 11
Description: Orthogonal xy-slicing through a MarShie-cleared diaphysis, two days after drill-hole injury in a CX3CR1-GFP x Cdh5-tdTomato/histone-GFP mouse. Cdh5-tdTom+ vessels is shown in magenta, autofluorescence signal of marrow and cortical structures at 488 nm excitation is displayed in grey. Nanobody Atto-647N enhanced GFP signals of CX3CR1-GFP myeloid cells are displayed in green and histone-linked-GFP of endothelial nuclei in white. Zoom-in highlights the altered morphology of CX3CR1-GFP+ myeloid cells forming a barrier-like structure around the drill-hole. 0.94 µm/pixel, z-step 3 µm. Image volume: 2 mm x 2 mm x 2 mm.
