# Supplementary figures and images for: MarShie: a clearing protocol for 3D analysis of single cells throughout the bone marrow at subcellular resolution
Source: Nat Commun. 2024 Feb 26;15:1764. doi: 10.1038/s41467-024-45827-6 (PMC10897183; doi:10.1038/s41467-024-45827-6)

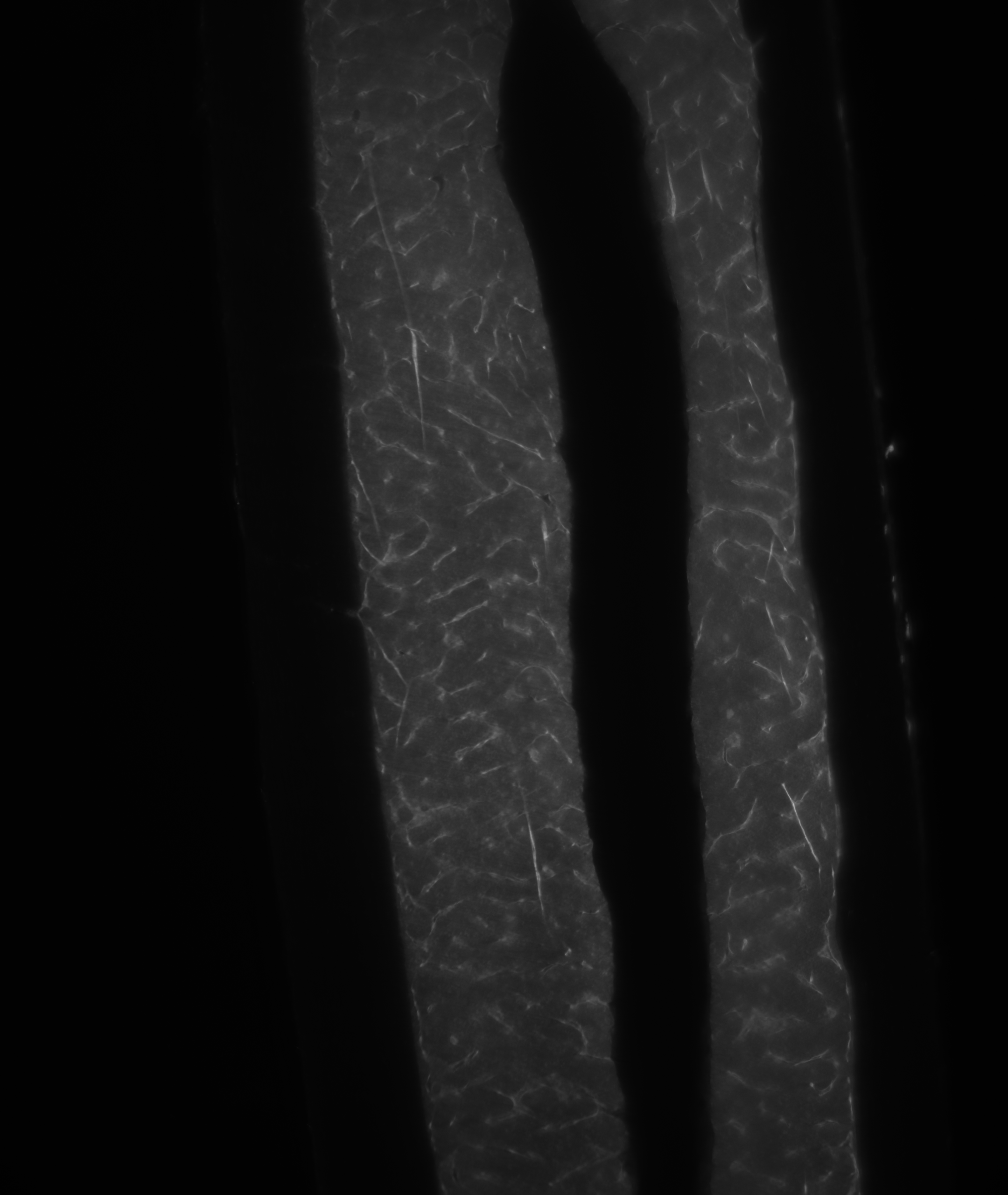

Supplement: Supplementary file 4 — Supplementary Software 1 [file 41467_2024_45827_MOESM4_ESM.zip › Mertens_Liebheit_Destriping_algorithm/Demo images destriped output/16-15-26_UltraII[02]_C01_xyz-Table Z0360.ome.tif]

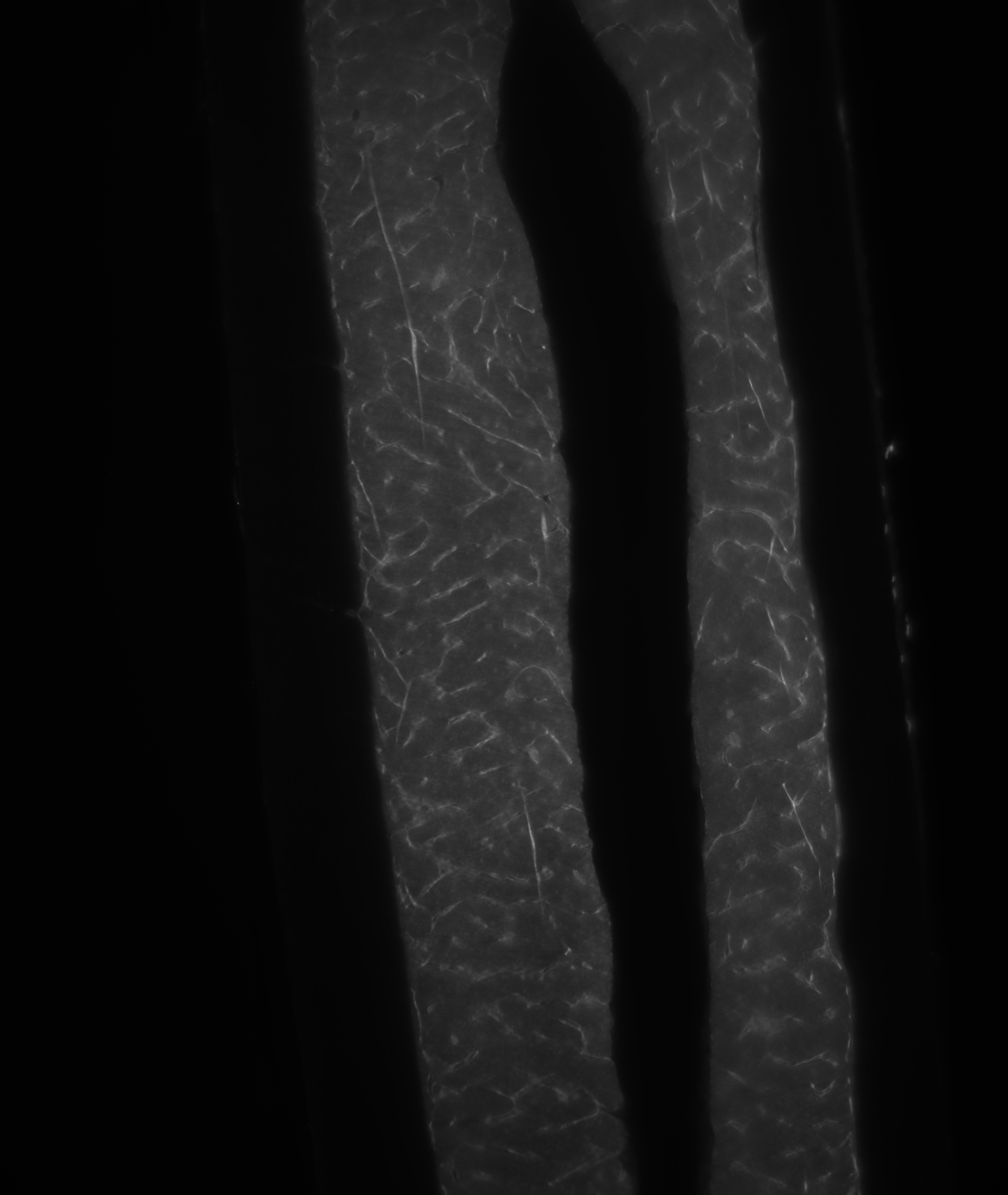

Supplement: Supplementary file 4 — Supplementary Software 1 [file 41467_2024_45827_MOESM4_ESM.zip › Mertens_Liebheit_Destriping_algorithm/Demo images destriped output/16-15-26_UltraII[02]_C01_xyz-Table Z0361.ome.tif]

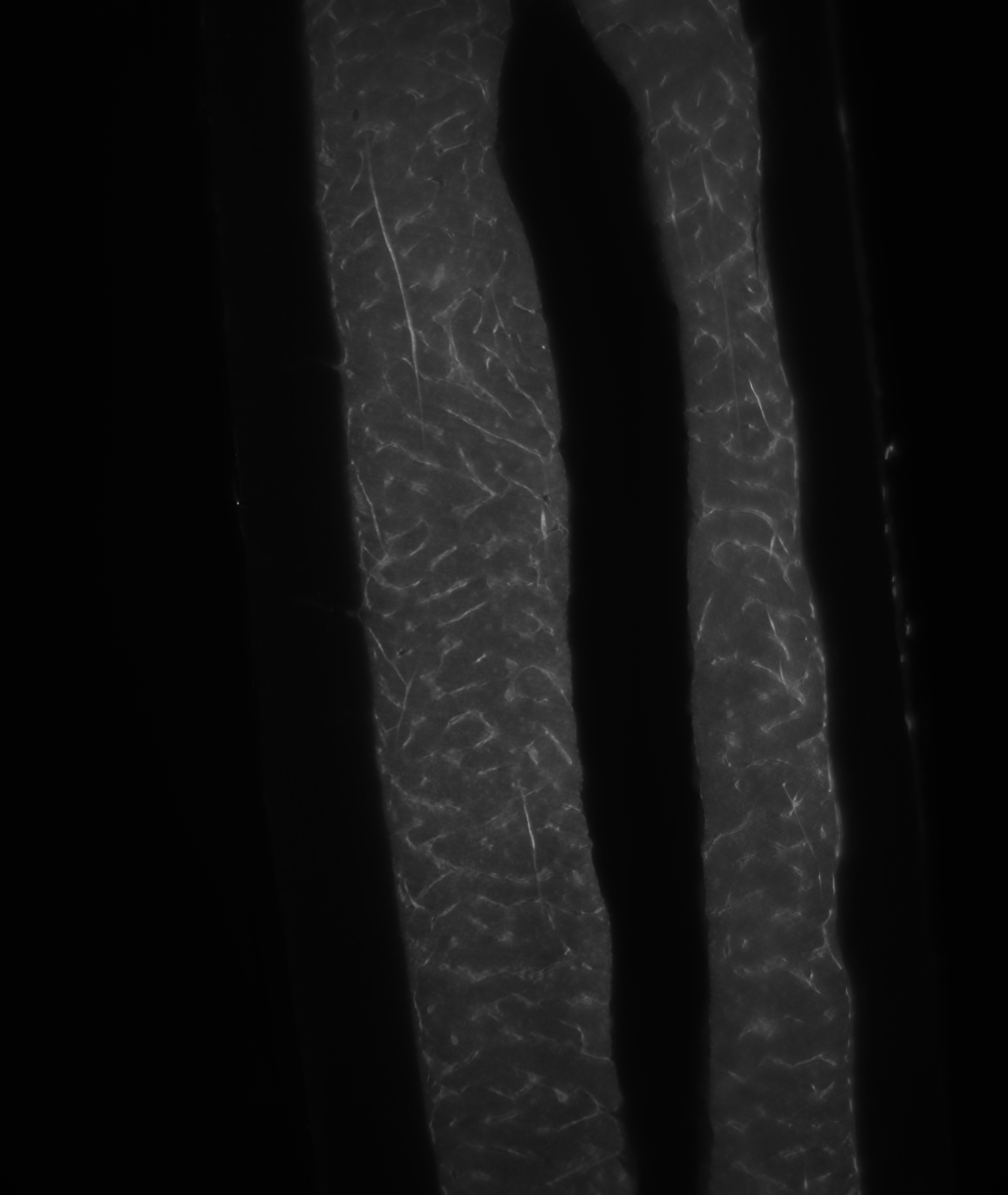

Supplement: Supplementary file 4 — Supplementary Software 1 [file 41467_2024_45827_MOESM4_ESM.zip › Mertens_Liebheit_Destriping_algorithm/Demo images destriped output/16-15-26_UltraII[02]_C01_xyz-Table Z0362.ome.tif]

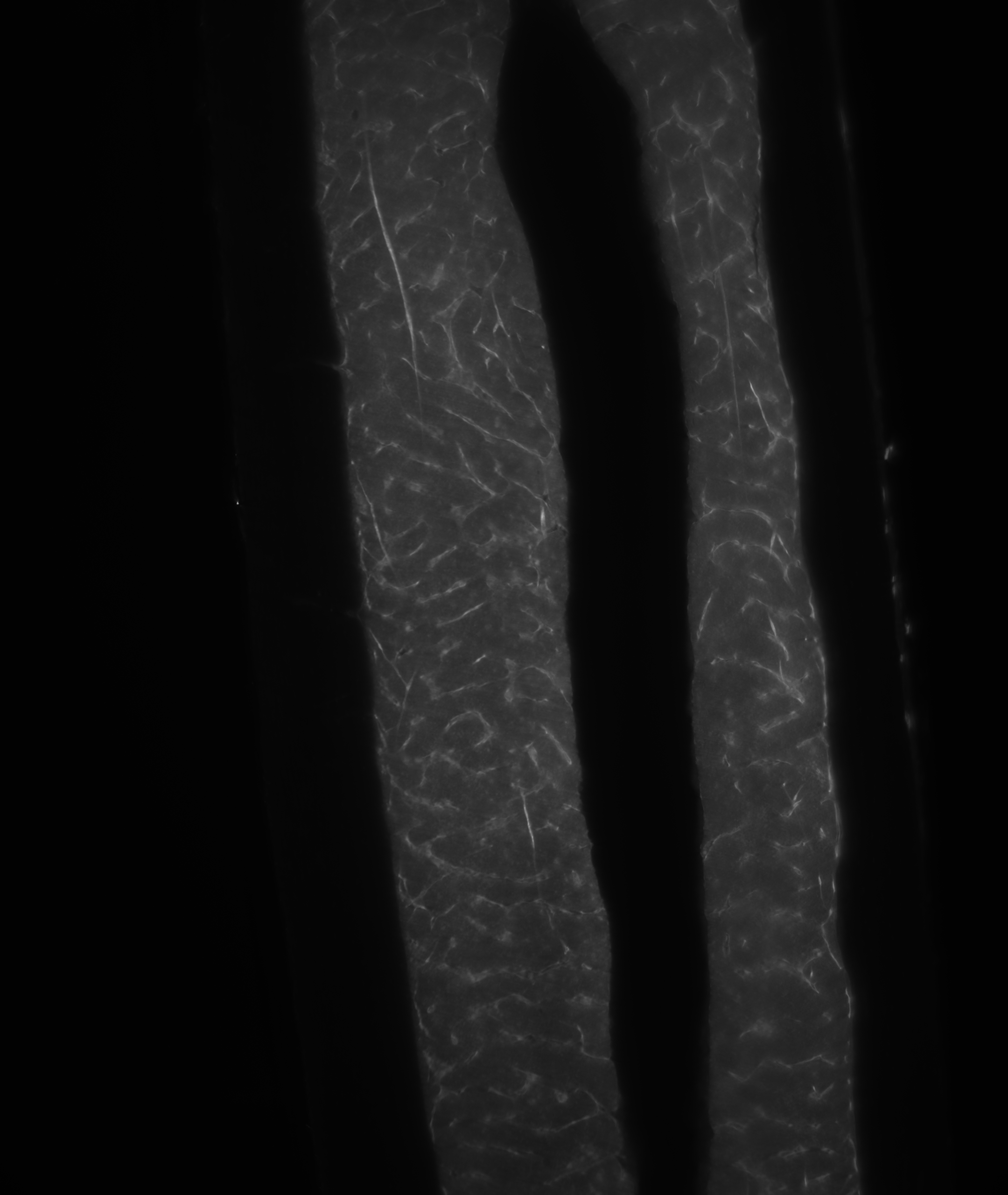

Supplement: Supplementary file 4 — Supplementary Software 1 [file 41467_2024_45827_MOESM4_ESM.zip › Mertens_Liebheit_Destriping_algorithm/Demo images destriped output/16-15-26_UltraII[02]_C01_xyz-Table Z0363.ome.tif]

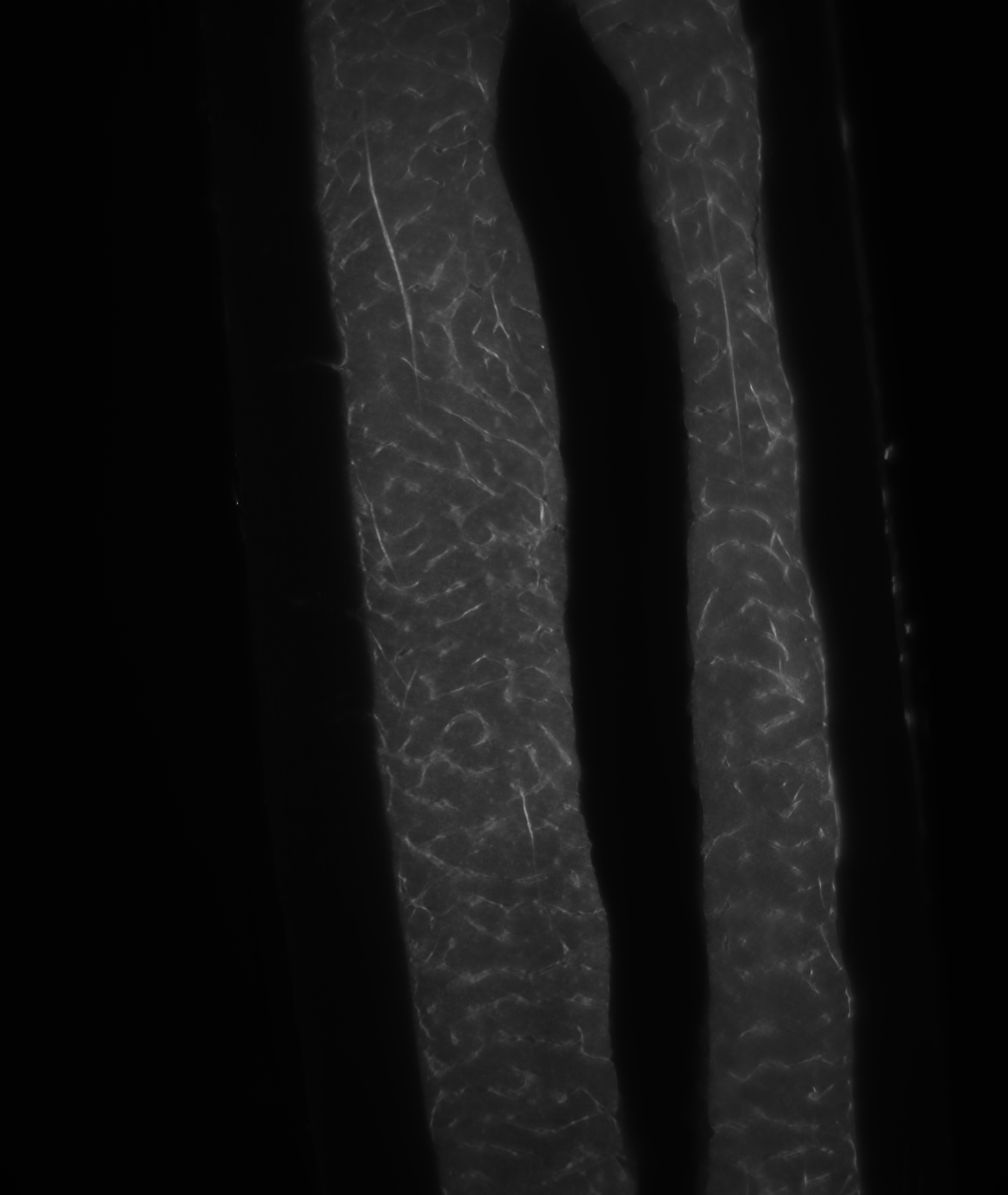

Supplement: Supplementary file 4 — Supplementary Software 1 [file 41467_2024_45827_MOESM4_ESM.zip › Mertens_Liebheit_Destriping_algorithm/Demo images destriped output/16-15-26_UltraII[02]_C01_xyz-Table Z0364.ome.tif]

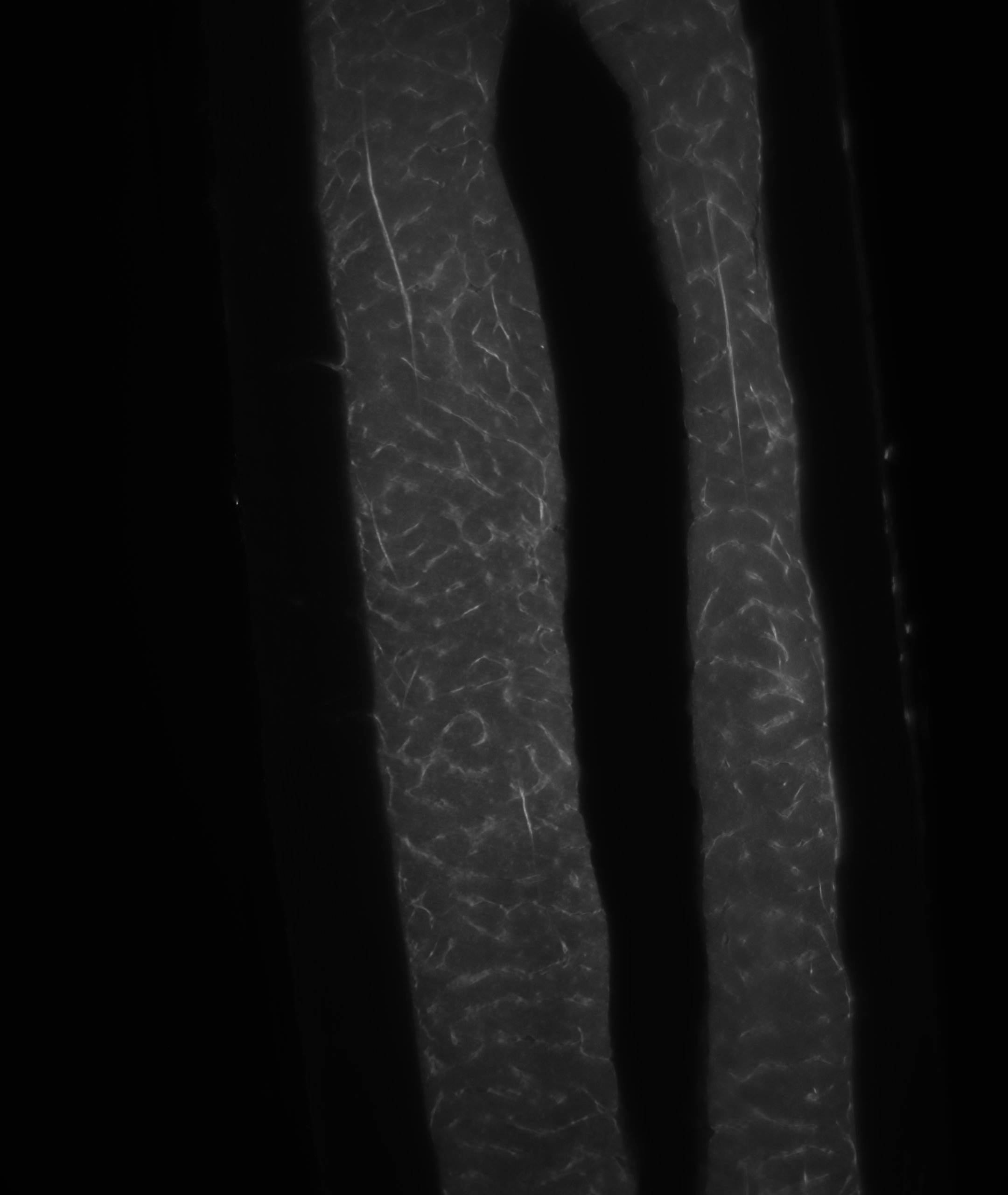

Supplement: Supplementary file 4 — Supplementary Software 1 [file 41467_2024_45827_MOESM4_ESM.zip › Mertens_Liebheit_Destriping_algorithm/Demo images destriped output/16-15-26_UltraII[02]_C01_xyz-Table Z0365.ome.tif]

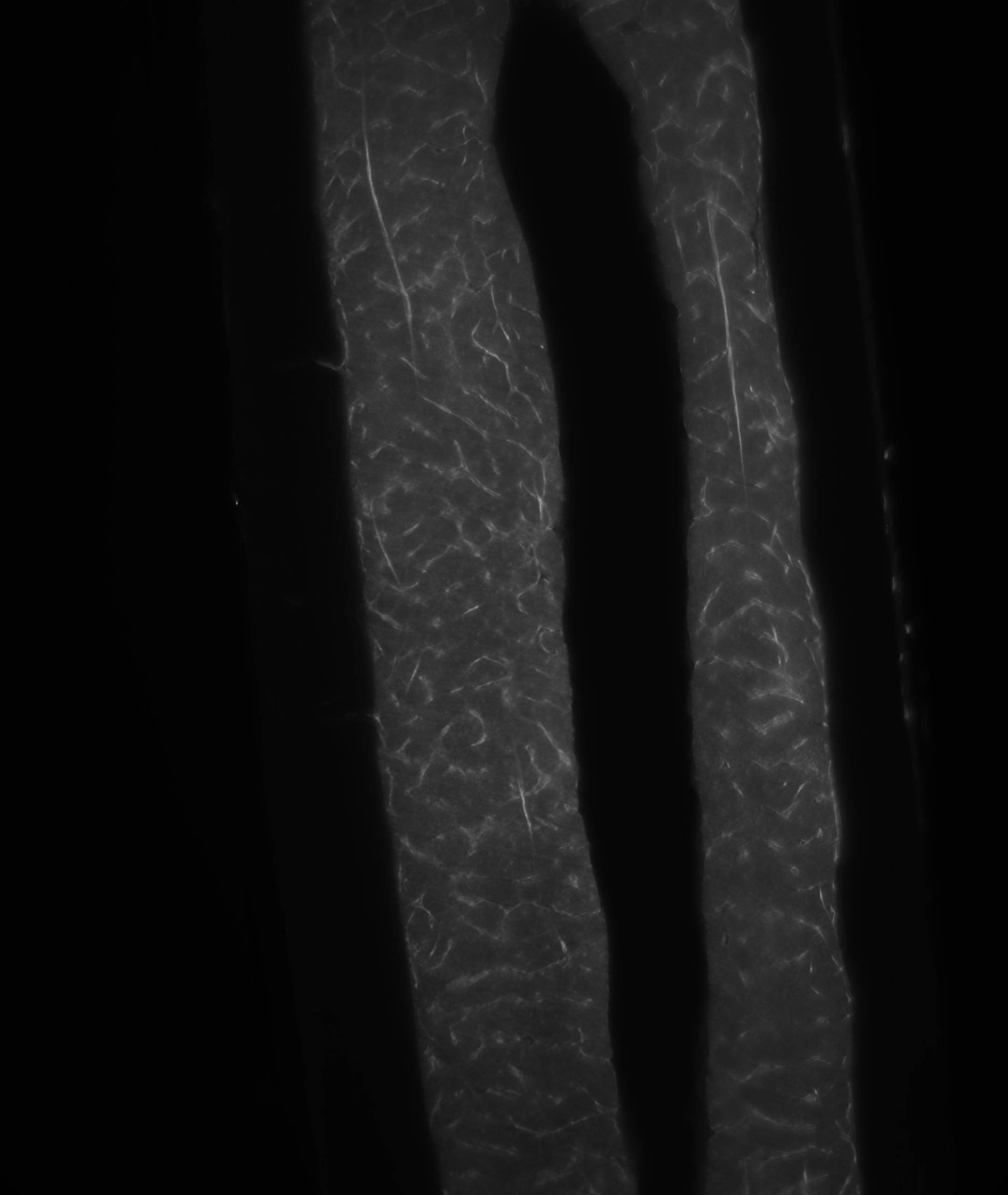

Supplement: Supplementary file 4 — Supplementary Software 1 [file 41467_2024_45827_MOESM4_ESM.zip › Mertens_Liebheit_Destriping_algorithm/Demo images destriped output/16-15-26_UltraII[02]_C01_xyz-Table Z0366.ome.tif]

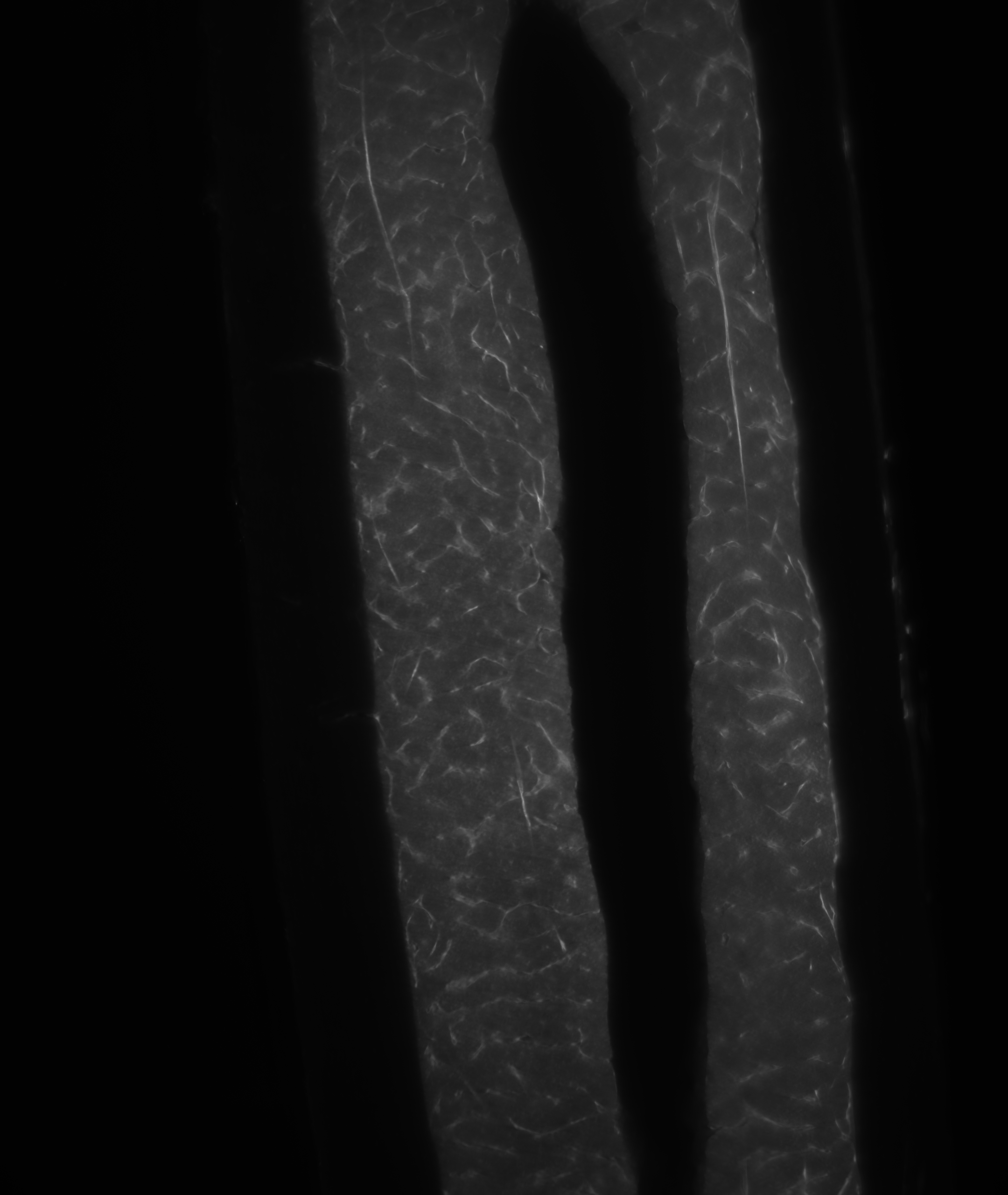

Supplement: Supplementary file 4 — Supplementary Software 1 [file 41467_2024_45827_MOESM4_ESM.zip › Mertens_Liebheit_Destriping_algorithm/Demo images destriped output/16-15-26_UltraII[02]_C01_xyz-Table Z0367.ome.tif]

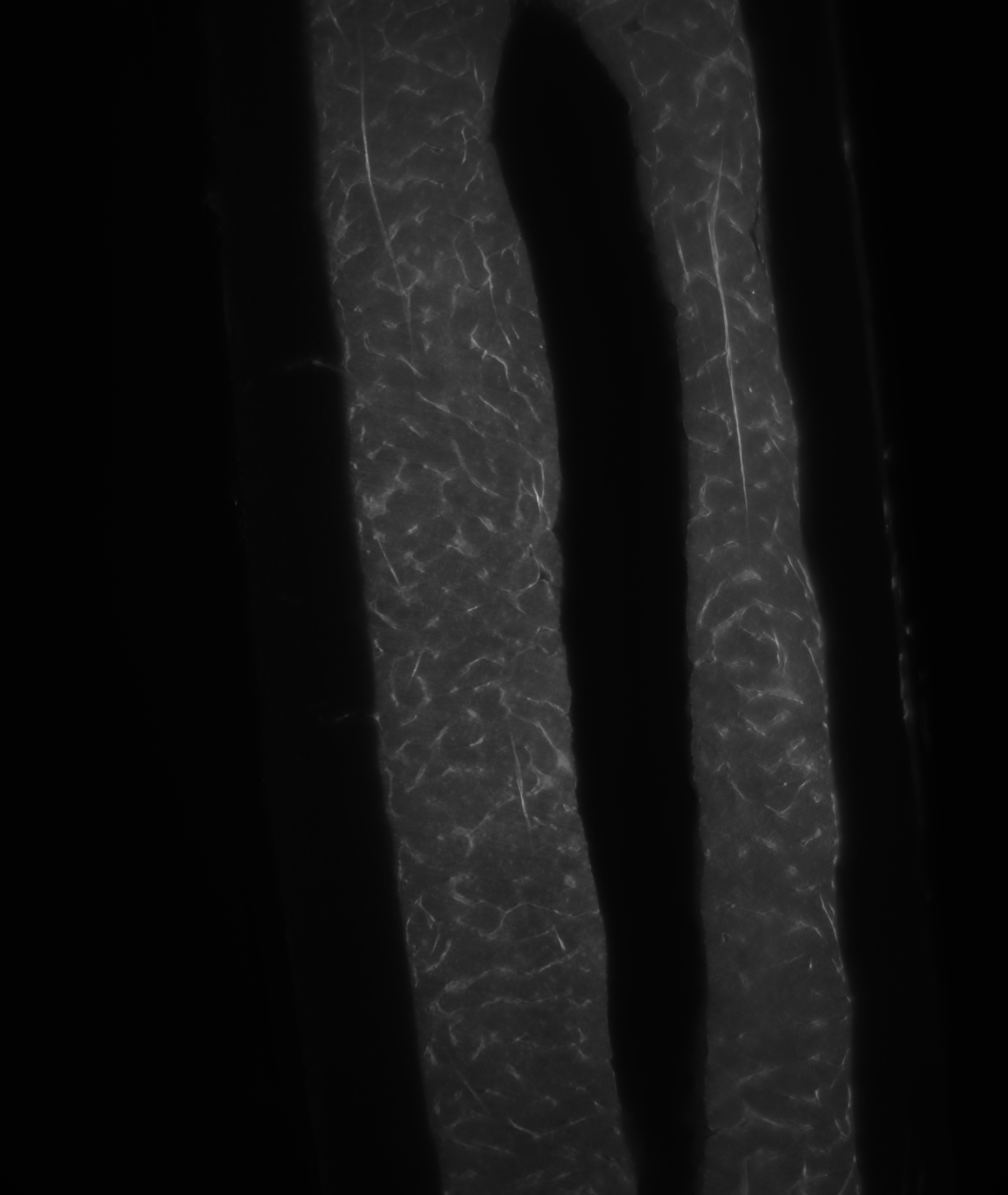

Supplement: Supplementary file 4 — Supplementary Software 1 [file 41467_2024_45827_MOESM4_ESM.zip › Mertens_Liebheit_Destriping_algorithm/Demo images destriped output/16-15-26_UltraII[02]_C01_xyz-Table Z0368.ome.tif]

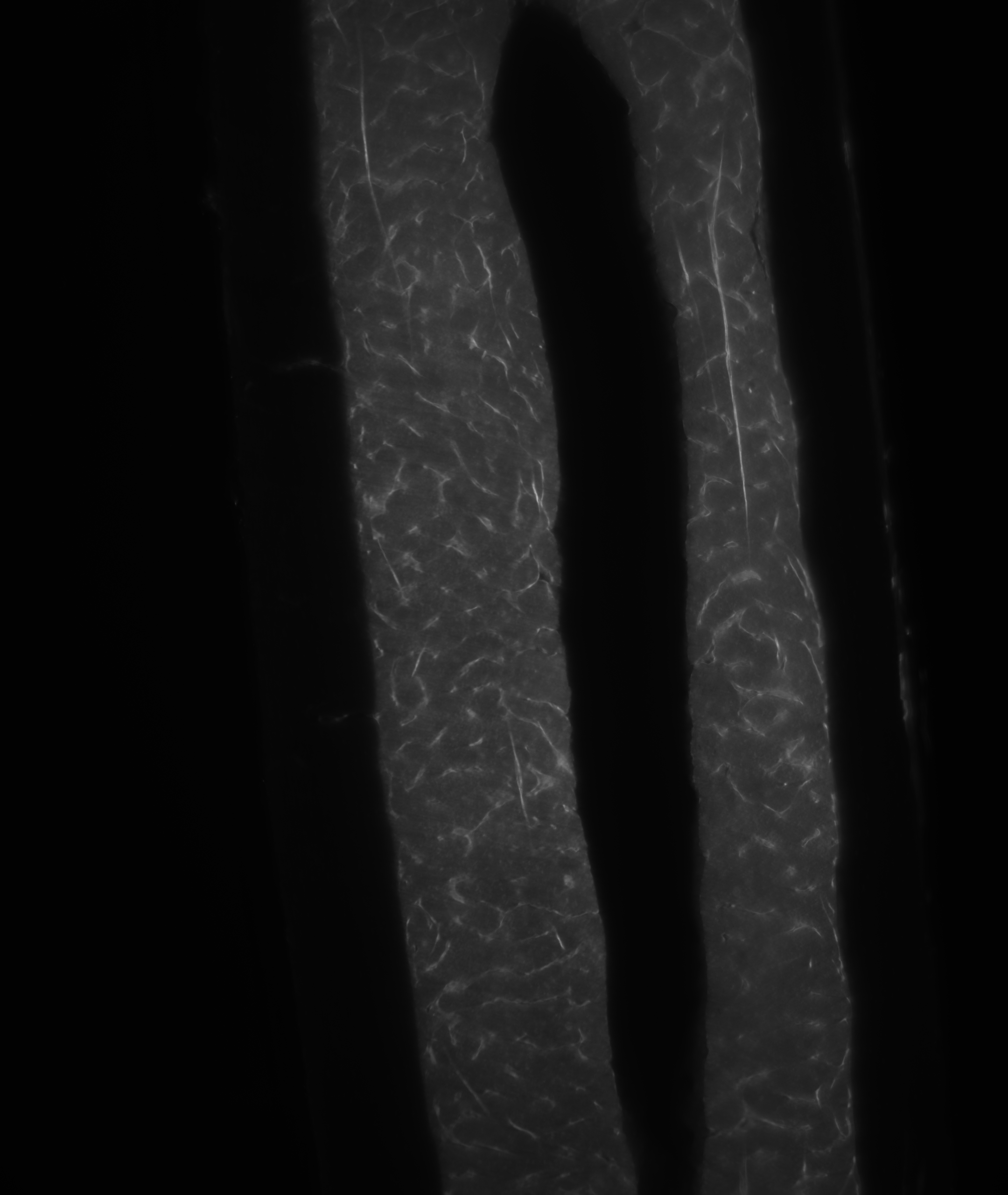

Supplement: Supplementary file 4 — Supplementary Software 1 [file 41467_2024_45827_MOESM4_ESM.zip › Mertens_Liebheit_Destriping_algorithm/Demo images destriped output/16-15-26_UltraII[02]_C01_xyz-Table Z0369.ome.tif]

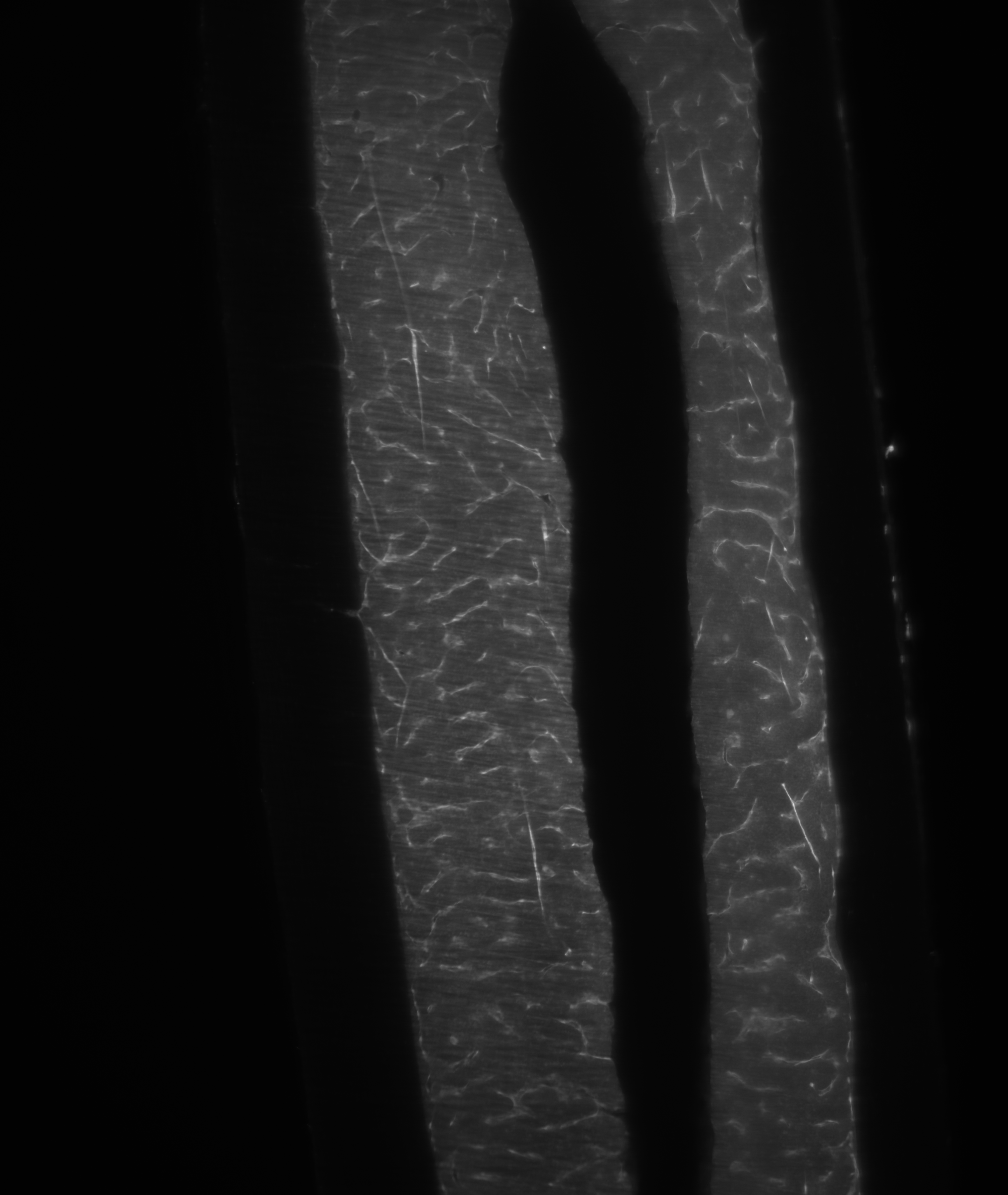

Supplement: Supplementary file 4 — Supplementary Software 1 [file 41467_2024_45827_MOESM4_ESM.zip › Mertens_Liebheit_Destriping_algorithm/Demo images raw data/16-15-26_UltraII[02]_C01_xyz-Table Z0360.ome.tif]

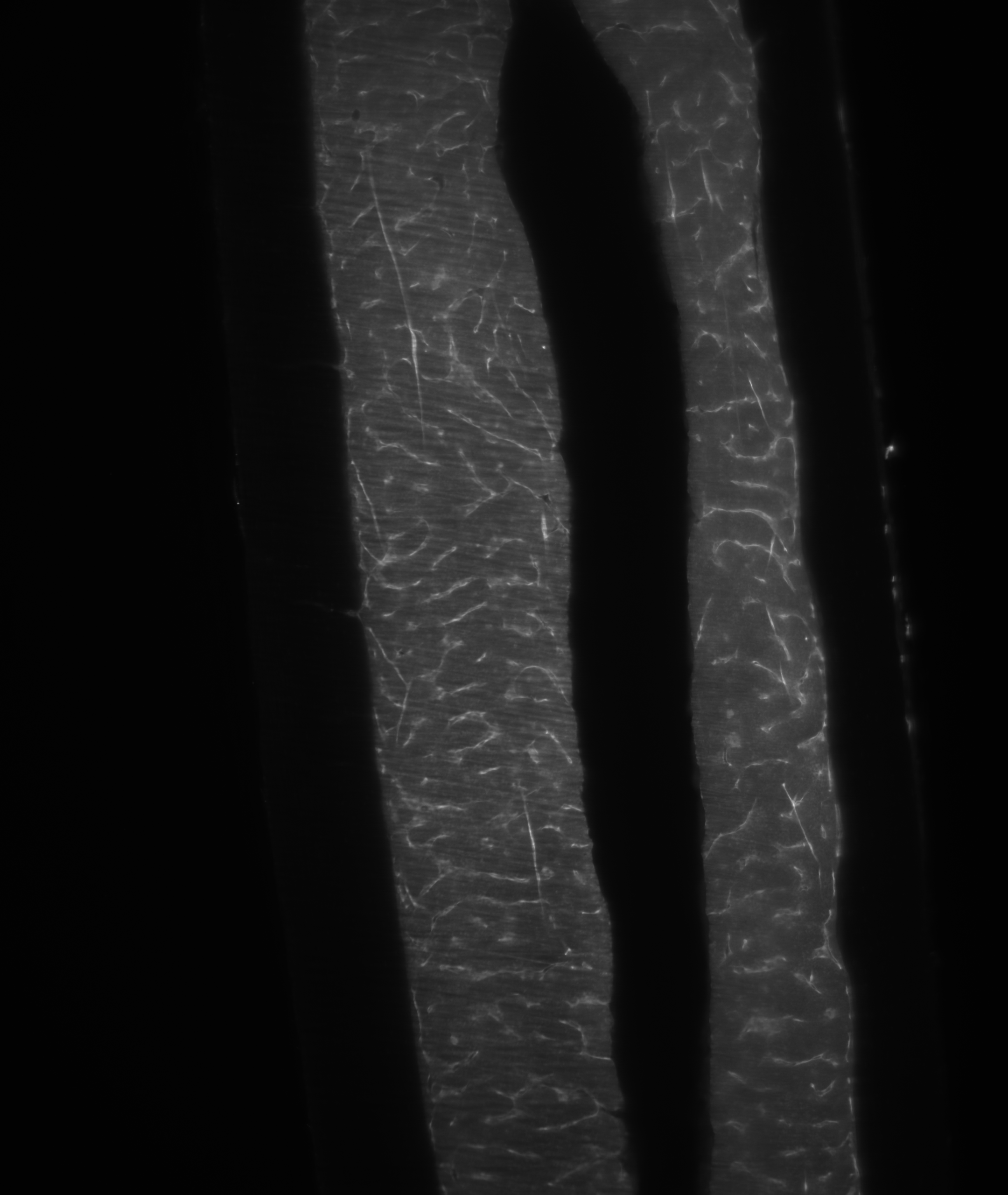

Supplement: Supplementary file 4 — Supplementary Software 1 [file 41467_2024_45827_MOESM4_ESM.zip › Mertens_Liebheit_Destriping_algorithm/Demo images raw data/16-15-26_UltraII[02]_C01_xyz-Table Z0361.ome.tif]

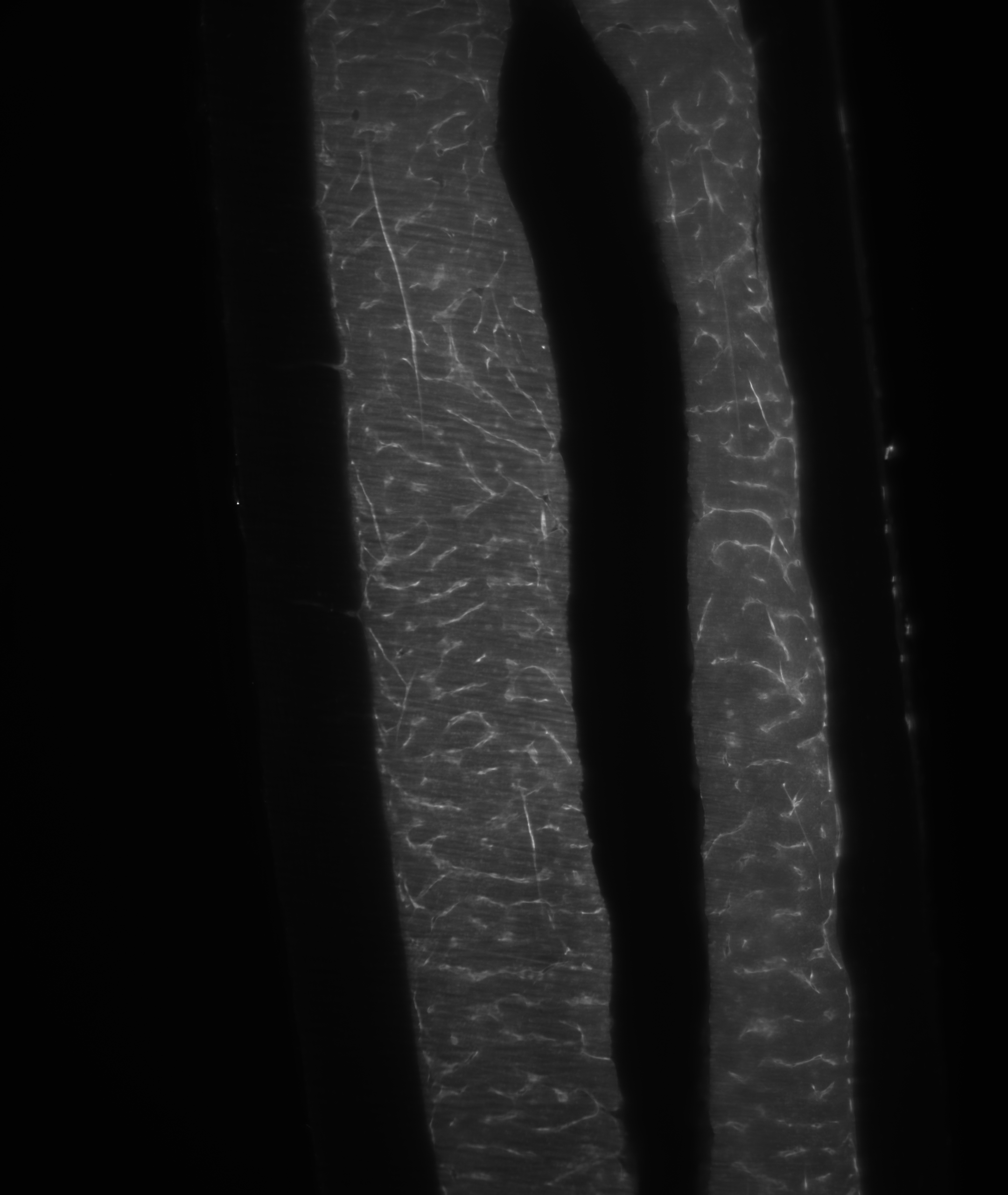

Supplement: Supplementary file 4 — Supplementary Software 1 [file 41467_2024_45827_MOESM4_ESM.zip › Mertens_Liebheit_Destriping_algorithm/Demo images raw data/16-15-26_UltraII[02]_C01_xyz-Table Z0362.ome.tif]

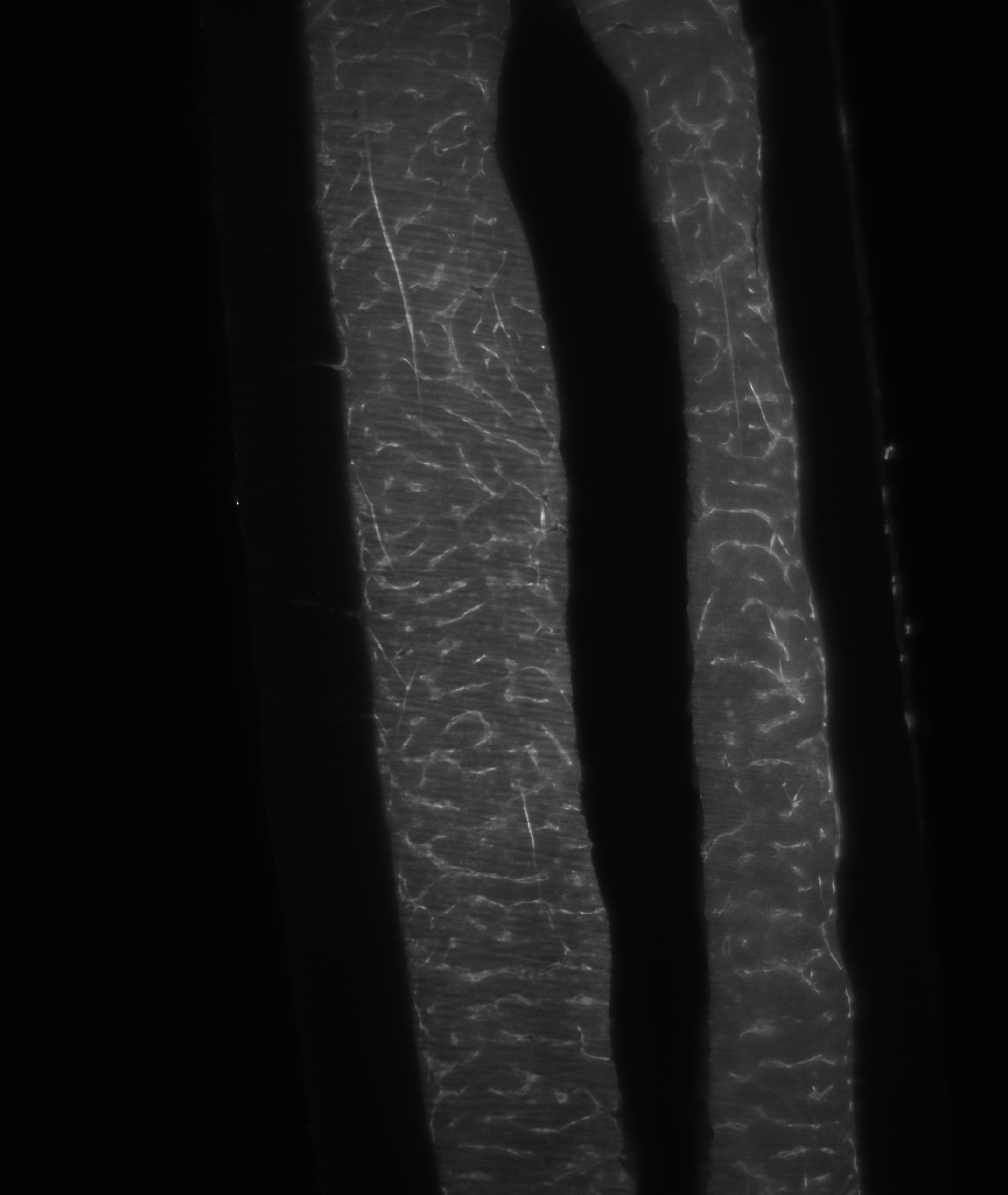

Supplement: Supplementary file 4 — Supplementary Software 1 [file 41467_2024_45827_MOESM4_ESM.zip › Mertens_Liebheit_Destriping_algorithm/Demo images raw data/16-15-26_UltraII[02]_C01_xyz-Table Z0363.ome.tif]

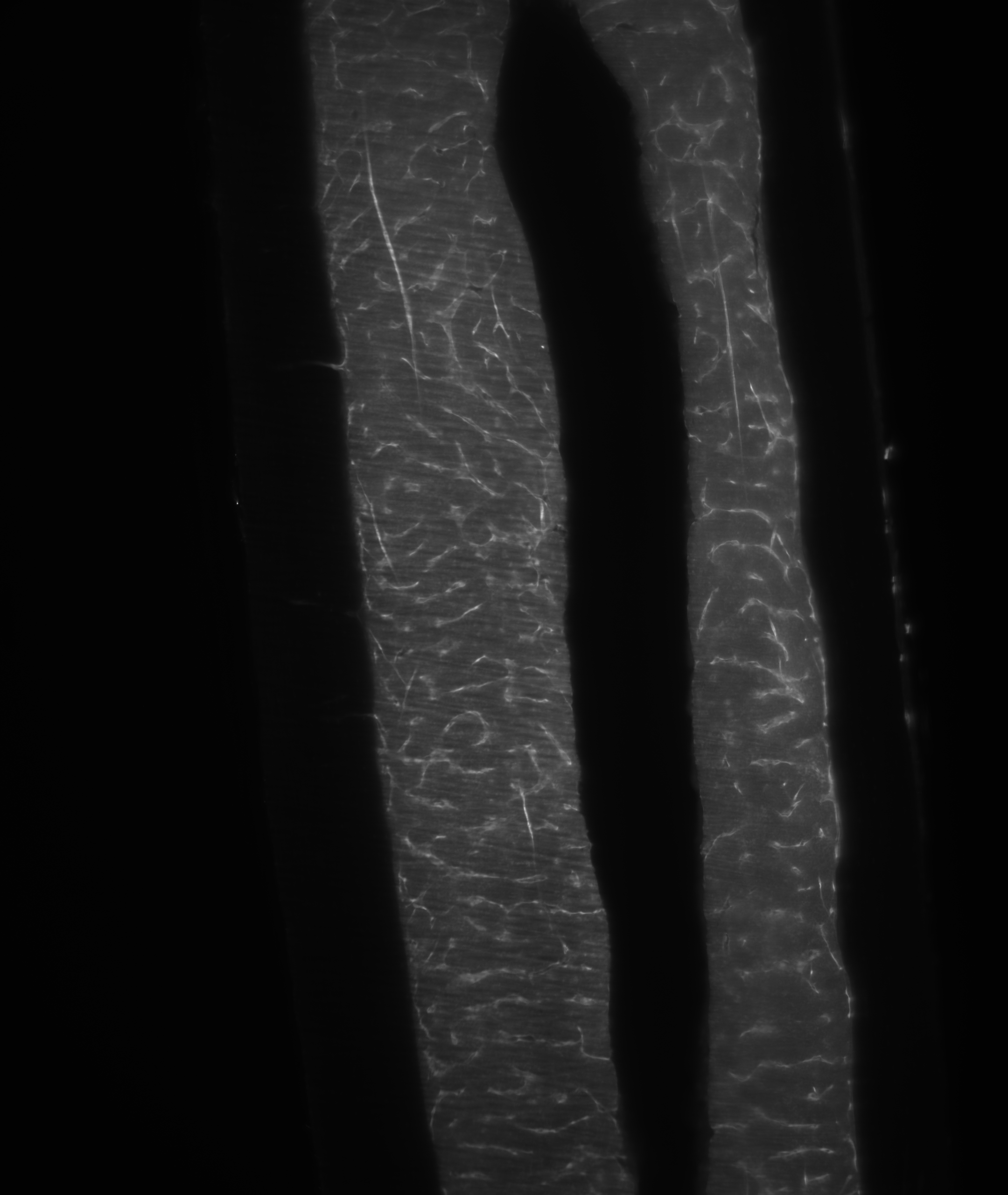

Supplement: Supplementary file 4 — Supplementary Software 1 [file 41467_2024_45827_MOESM4_ESM.zip › Mertens_Liebheit_Destriping_algorithm/Demo images raw data/16-15-26_UltraII[02]_C01_xyz-Table Z0364.ome.tif]

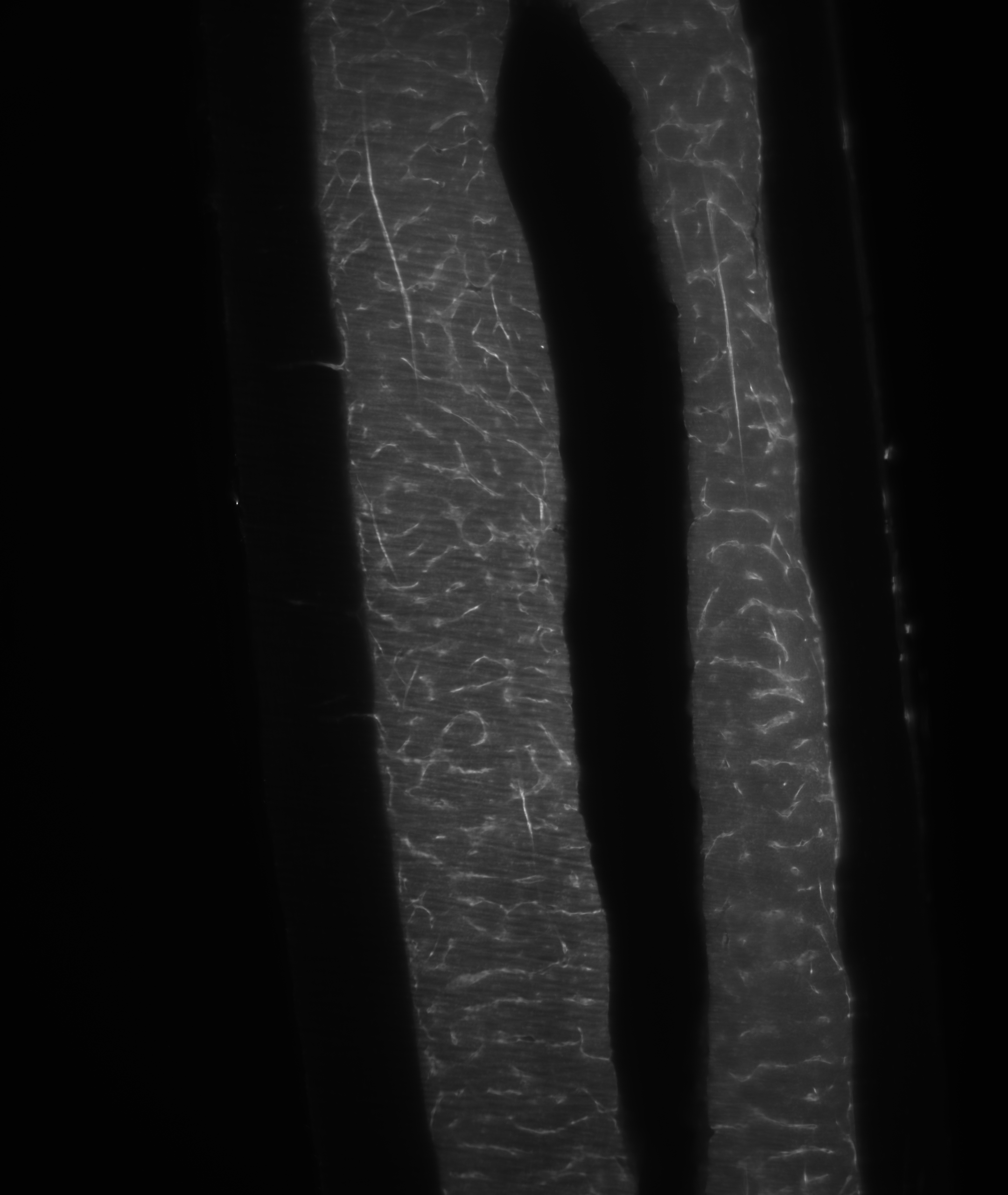

Supplement: Supplementary file 4 — Supplementary Software 1 [file 41467_2024_45827_MOESM4_ESM.zip › Mertens_Liebheit_Destriping_algorithm/Demo images raw data/16-15-26_UltraII[02]_C01_xyz-Table Z0365.ome.tif]

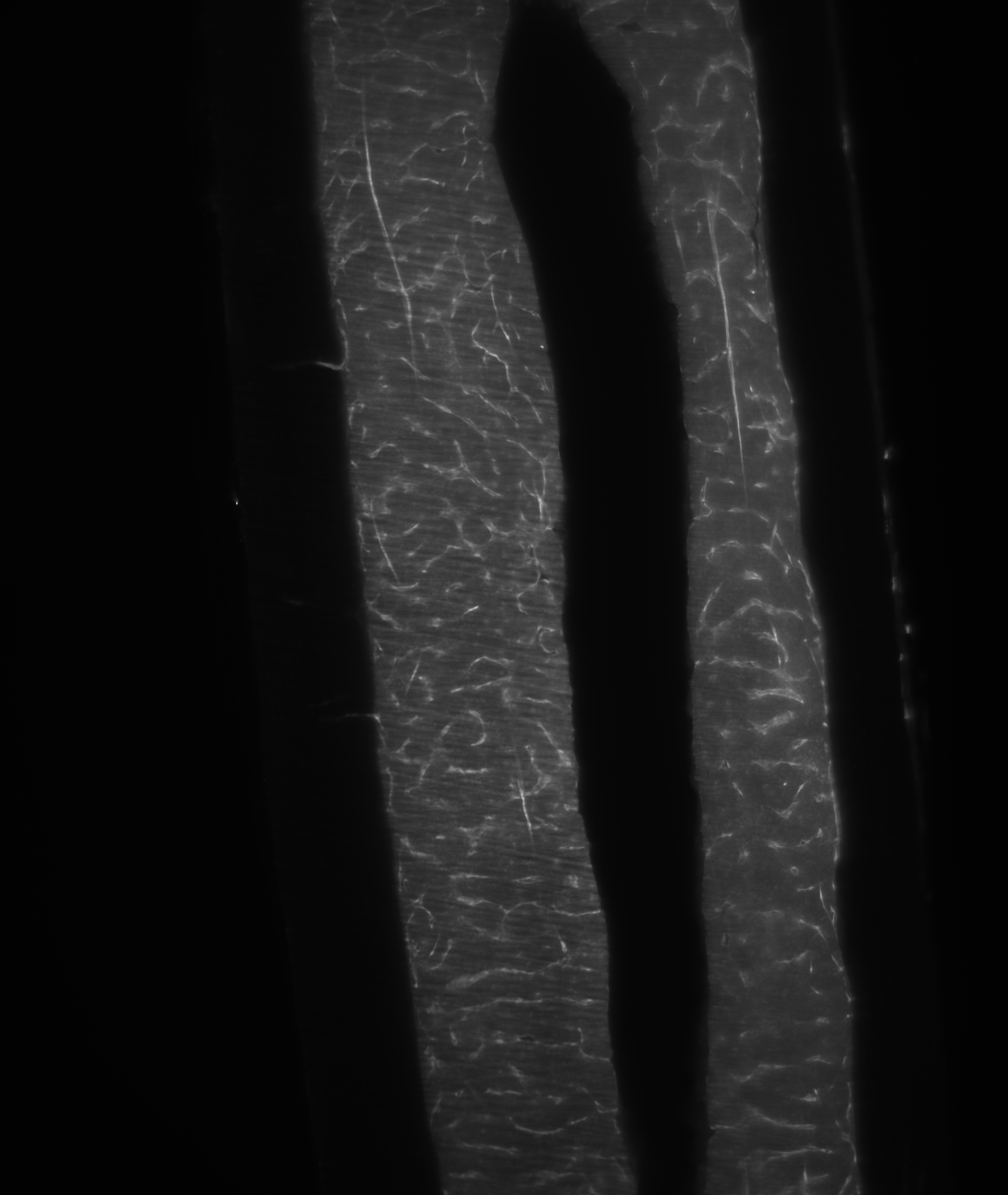

Supplement: Supplementary file 4 — Supplementary Software 1 [file 41467_2024_45827_MOESM4_ESM.zip › Mertens_Liebheit_Destriping_algorithm/Demo images raw data/16-15-26_UltraII[02]_C01_xyz-Table Z0366.ome.tif]

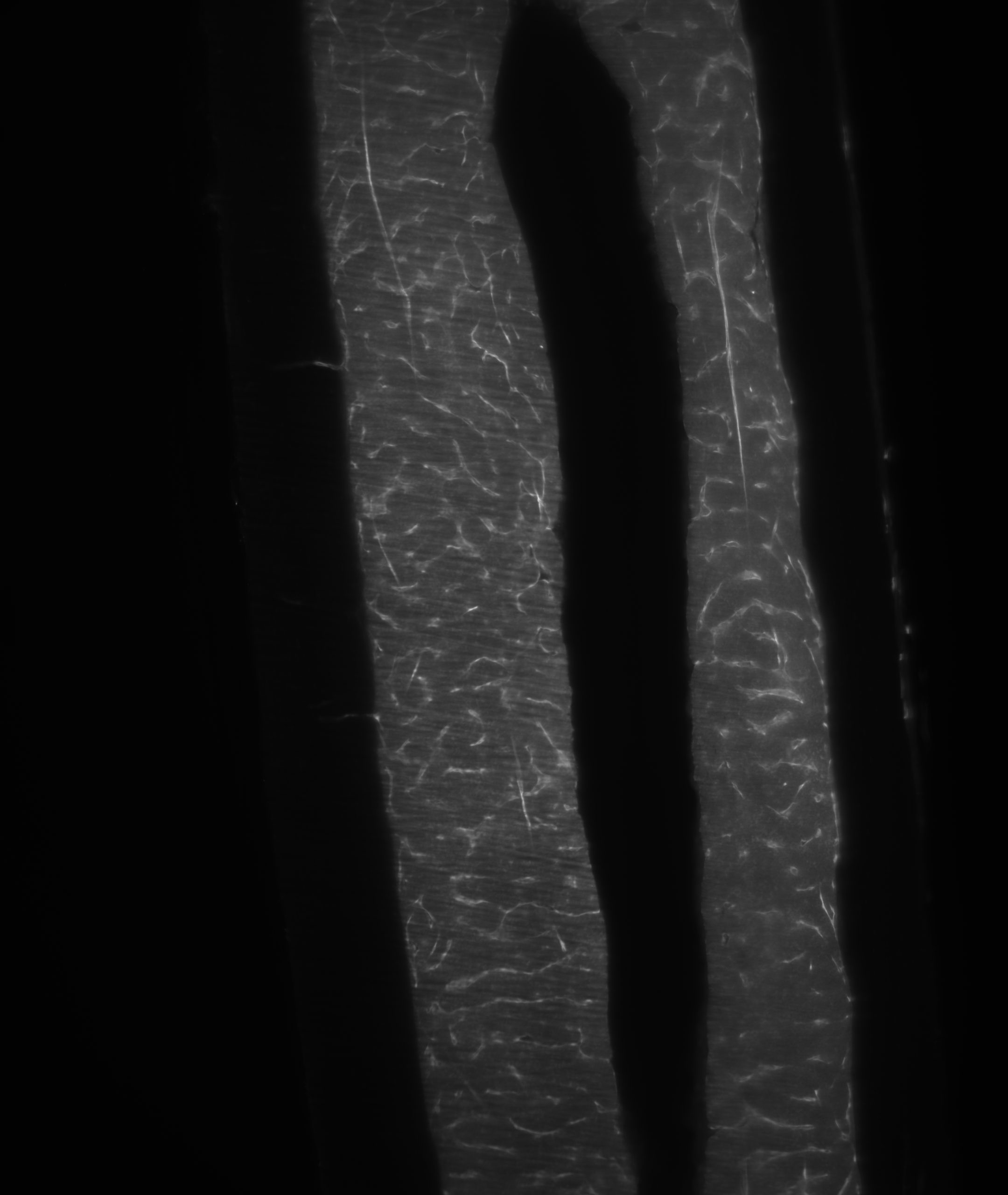

Supplement: Supplementary file 4 — Supplementary Software 1 [file 41467_2024_45827_MOESM4_ESM.zip › Mertens_Liebheit_Destriping_algorithm/Demo images raw data/16-15-26_UltraII[02]_C01_xyz-Table Z0367.ome.tif]

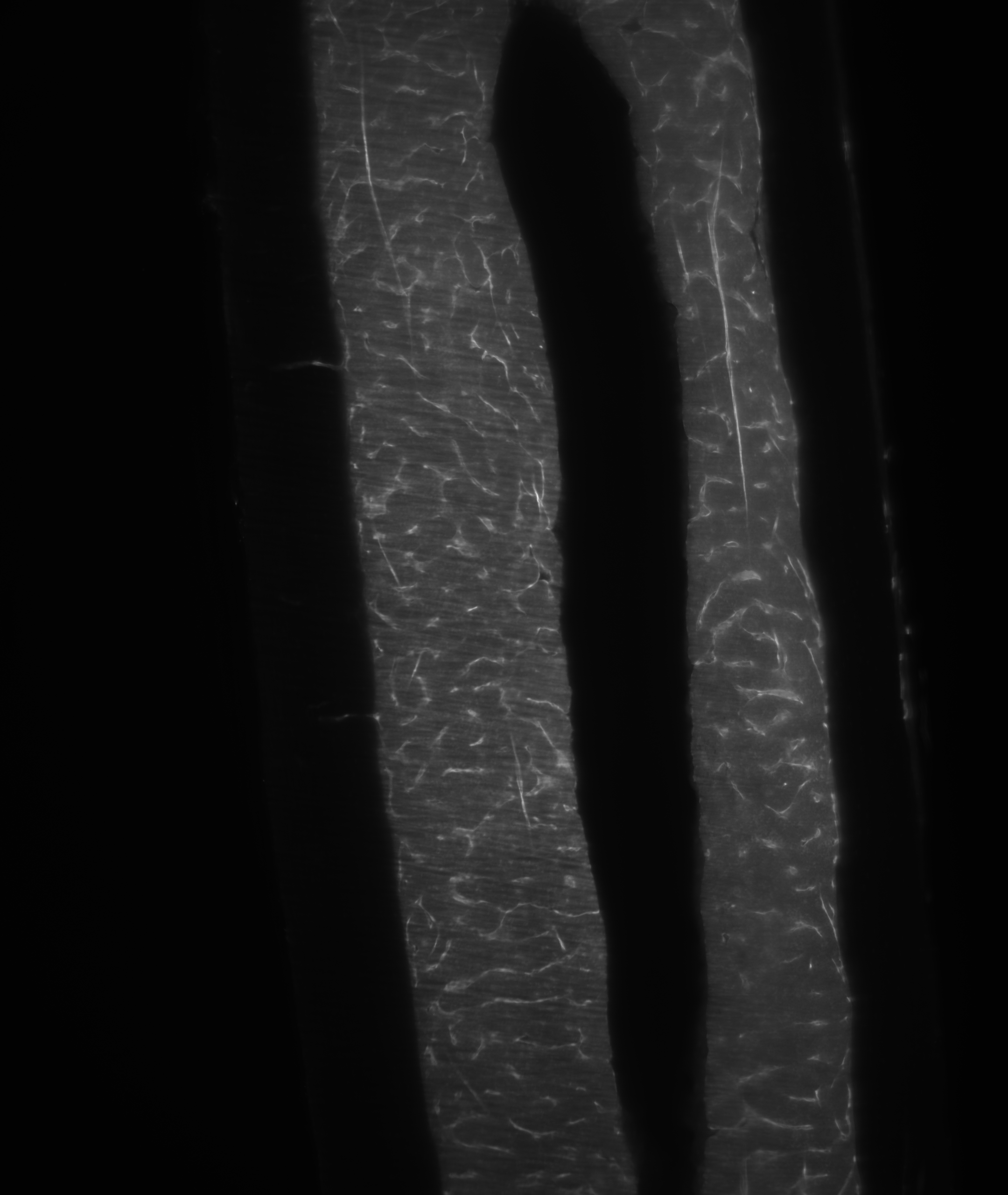

Supplement: Supplementary file 4 — Supplementary Software 1 [file 41467_2024_45827_MOESM4_ESM.zip › Mertens_Liebheit_Destriping_algorithm/Demo images raw data/16-15-26_UltraII[02]_C01_xyz-Table Z0368.ome.tif]

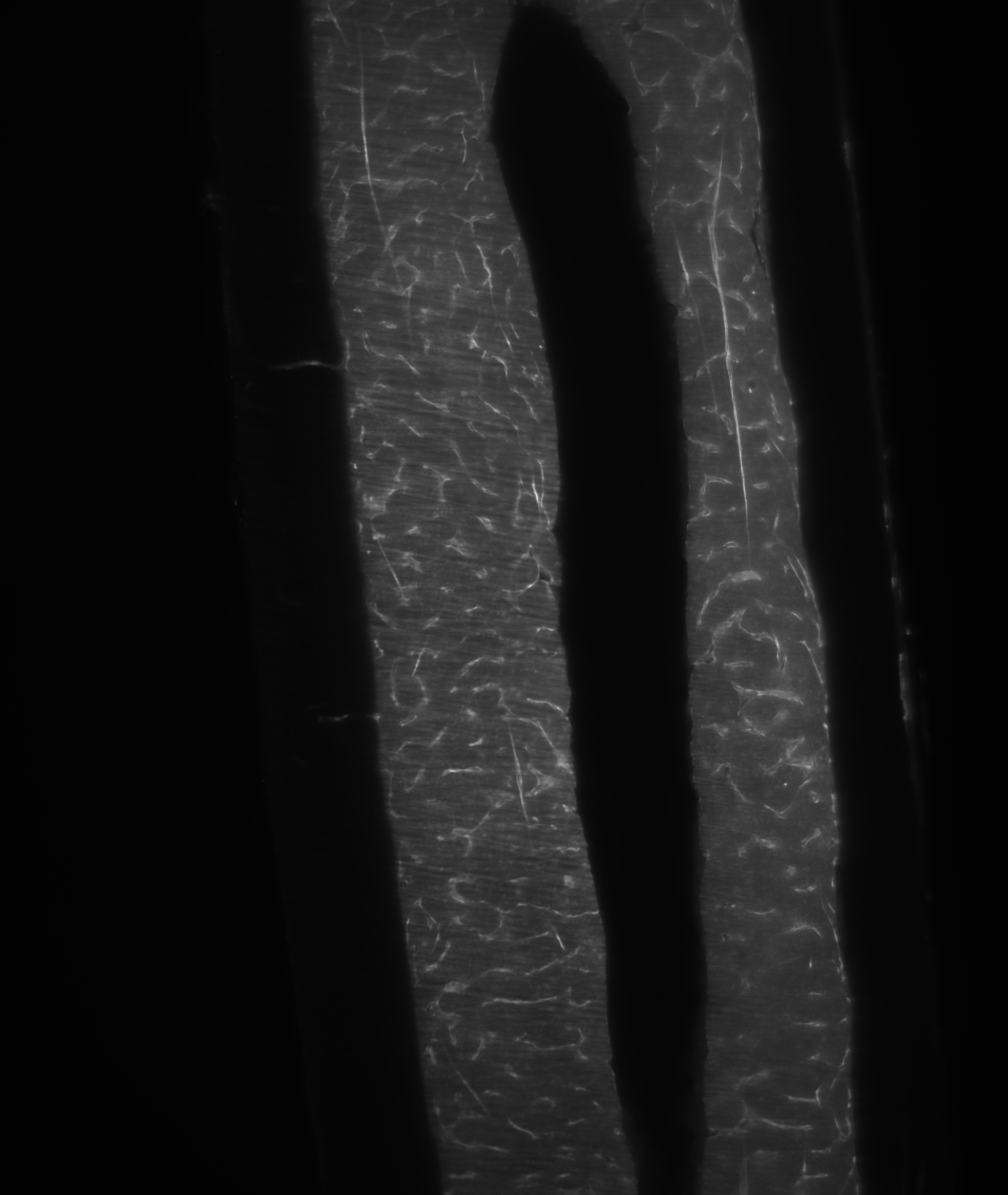

Supplement: Supplementary file 4 — Supplementary Software 1 [file 41467_2024_45827_MOESM4_ESM.zip › Mertens_Liebheit_Destriping_algorithm/Demo images raw data/16-15-26_UltraII[02]_C01_xyz-Table Z0369.ome.tif]
