# Supplementary material for: MarShie: a clearing protocol for 3D analysis of single cells throughout the bone marrow at subcellular resolution
Source: Nat Commun. 2024 Feb 26;15:1764. doi: 10.1038/s41467-024-45827-6 (PMC10897183; doi:10.1038/s41467-024-45827-6)
Supplement: Supplementary file 16 — Reporting Summary [file 41467_2024_45827_MOESM16_ESM.pdf]

nature portfolio

Corresponding author(s): Anja Erika Hauser

Last updated by author(s): 18.12.2023

Reporting Summary

Nature Portfolio wishes to improve the reproducibility of the work that we publish. This form provides structure for consistency and transparency in reporting. For further information on Nature Portfolio policies, see our Editorial Policies and the Editorial Policy Checklist.

Please do not complete any field with "not applicable" or n/a. Refer to the help text for what text to use if an item is not relevant to your study.
For final submission: please carefully check your responses for accuracy; you will not be able to make changes later.

Statistics

For all statistical analyses, confirm that the following items are present in the figure legend, table legend, main text, or Methods section.

n/a	Confirmed	
☐	☒	The exact sample size (*n*) for each experimental group/condition, given as a discrete number and unit of measurement
☐	☒	A statement on whether measurements were taken from distinct samples or whether the same sample was measured repeatedly
☐	☒	The statistical test(s) used AND whether they are one- or two-sided<br>*Only common tests should be described solely by name; describe more complex techniques in the Methods section.*
☒	☐	A description of all covariates tested
☒	☐	A description of any assumptions or corrections, such as tests of normality and adjustment for multiple comparisons
☐	☒	A full description of the statistical parameters including central tendency (e.g. means) or other basic estimates (e.g. regression coefficient) AND variation (e.g. standard deviation) or associated estimates of uncertainty (e.g. confidence intervals)
☐	☒	For null hypothesis testing, the test statistic (e.g. *F*, *t*, *r*) with confidence intervals, effect sizes, degrees of freedom and *P* value noted<br>*Give P values as exact values whenever suitable.*
☒	☐	For Bayesian analysis, information on the choice of priors and Markov chain Monte Carlo settings
☒	☐	For hierarchical and complex designs, identification of the appropriate level for tests and full reporting of outcomes
☒	☐	Estimates of effect sizes (e.g. Cohen's *d*, Pearson's *r*), indicating how they were calculated

Our web collection on statistics for biologists contains articles on many of the points above.

Software and code

Policy information about availability of computer code

Data collection	Light Sheet data were colllected on an Ultramicroscope II (LaVision/Miltenyi Biotec) with ImSpector Pro software v073. Spectroscopic data were acquired using an OceanInsight SR-4VN500-25 Spectrometer.
Data analysis	Analysis was performed in Imaris, Imaris File Converter and Imaris Stitcher v9.9.1, FIJI ImageJ v1.53c (NIH), GraphPad Prism v5 (GraphPad Software Inc.), Origin v9.0 (Originlab). The de-striping algorithm is publicly available, doi:10.5281/zenodo 10374405

For manuscripts utilizing custom algorithms or software that are central to the research but not yet described in published literature, software must be made available to editors and reviewers. We strongly encourage code deposition in a community repository (e.g. GitHub). See the Nature Portfolio guidelines for submitting code & software for further information.

Data

Policy information about availability of data

All manuscripts must include a data availability statement. This statement should provide the following information, where applicable:
- Accession codes, unique identifiers, or web links for publicly available datasets
- A description of any restrictions on data availability
- For clinical datasets or third party data, please ensure that the statement adheres to our policy

The raw imaging data are under restricted access because of their large size, access can be obtained upon request to the corresponding author. Requests will be fulfilled within 2 weeks. Source data are provided with the paper.

Research involving human participants, their data, or biological material

Policy information about studies with human participants or human data. See also policy information about sex, gender (identity/presentation), and sexual orientation and race, ethnicity and racism.

Reporting on sex and gender	N/A
Reporting on race, ethnicity, or other socially relevant groupings	N/A
Population characteristics	N/A
Recruitment	N/A
Ethics oversight	N/A

Note that full information on the approval of the study protocol must also be provided in the manuscript.

Field-specific reporting

Please select the one below that is the best fit for your research. If you are not sure, read the appropriate sections before making your selection.

[X] Life sciences [ ] Behavioural & social sciences [ ] Ecological, evolutionary & environmental sciences

For a reference copy of the document with all sections, see nature.com/documents/nr-reporting-summary-flat.pdf

Life sciences study design

All studies must disclose on these points even when the disclosure is negative.

Sample size	Sample sizes were chosen based on preliminary findings and pilot experiments.
Data exclusions	No data were excluded from the analysis.
Replication	We conducted multiple independent experiments for all conditions tested, details are given in the figure legends.
Randomization	All animals and samples were randomly subjected to the respective procedures.
Blinding	As the main purpose of this study was to establish a novel method, blinding was not performed, except for the data shown in Fig. 4C and 4H.

Behavioural & social sciences study design

All studies must disclose on these points even when the disclosure is negative.

Study description	
Research sample	
Sampling strategy	
Data collection	
Timing	
Data exclusions	
Non-participation	
Randomization	

Ecological, evolutionary & environmental sciences study design

All studies must disclose on these points even when the disclosure is negative.

Study description

Research sample

Sampling strategy

Data collection

Timing and spatial scale

Data exclusions

Reproducibility

Randomization

Blinding

Did the study involve field work? [ ] Yes [X] No

Field work, collection and transport

Field conditions

Location

Access & import/export

Disturbance

Reporting for specific materials, systems and methods

We require information from authors about some types of materials, experimental systems and methods used in many studies. Here, indicate whether each material, system or method listed is relevant to your study. If you are not sure if a list item applies to your research, read the appropriate section before selecting a response.

Materials & experimental systems

n/a	Involved in the study	
[ ]	[X]	Antibodies
[X]	[ ]	Eukaryotic cell lines
[X]	[ ]	Palaeontology and archaeology
[ ]	[X]	Animals and other organisms
[X]	[ ]	Clinical data
[X]	[ ]	Dual use research of concern
[X]	[ ]	Plants

Methods

n/a	Involved in the study	
[X]	[ ]	ChIP-seq
[X]	[ ]	Flow cytometry
[X]	[ ]	MRI-based neuroimaging

Antibodies

Antibodies used: Antibodies used for i.v. staining were 15 µg CD31 conjugated to Alexa Fluor 647 (BioLegend, Cat.# 102516, Clone: MEC13.3) and 20 µg Anti-Mo CD169 conjugated to eFluor 660 (Invitrogen, Cat.# 2211012, Clone: SER-4). Nanoboostering was performed using 8-12 µg of anti-GFP-Atto647N, ChromoTek GmbH, Cat. # gba647N-100.

Validation: Validation data and statements can be found on the manufacturers' websites. Whole-mount labeling using the anti-GFP-ATTO647N nanobodies was performed based on published methods (PMID: 30598527 and PMID:36697871).

Eukaryotic cell lines

Policy information about cell lines and Sex and Gender in Research

Cell line source(s)

Authentication

Mycoplasma contamination

Commonly misidentified lines
(See ICLAC register)

Palaeontology and Archaeology

Specimen provenance

Specimen deposition

Dating methods

☐ Tick this box to confirm that the raw and calibrated dates are available in the paper or in Supplementary Information.

Ethics oversight

Note that full information on the approval of the study protocol must also be provided in the manuscript.

Animals and other research organisms

Policy information about studies involving animals; ARRIVE guidelines recommended for reporting animal research, and Sex and Gender in Research

Laboratory animals: Mice were kept under specific pathogen free conditions, maintaining a 12-hour light/dark cycle with ambient temperature set to 22 ± 2 °C and air humidity 55 ± 10% rH, in the animal facilities of the Charité Crossover (CCO) facility and/or the DRFZ. Food and autoclaved water were provided ad libitum and animals were housed in individually ventilated cages containing an enriched environment. Animal experiments were conducted following the 3R Principles. Mice were handled using tunnels to reduce stress. Strain information is included at the end of this document due to space limitations..

Wild animals: This study did not include wild animals.

Reporting on sex: Due to our research interest in sex-specific osteo-immunological phenotypes in aged animals, we chose to work with female mice, except for the experiments involving the Fucci 639 strain, where male mice were not available to us.

Field-collected samples: No field-collected samples were used in this study.

Ethics oversight: All experimental procedures involving animals were carried out following approval of the study protocols by the Landesamt für Gesundheit und Soziales Berlin, animal license numbers G302/17 & G0025-22).

Note that full information on the approval of the study protocol must also be provided in the manuscript.

Clinical data

Policy information about clinical studies

All manuscripts should comply with the ICMJE guidelines for publication of clinical research and a completed CONSORT checklist must be included with all submissions.

Clinical trial registration

Study protocol

Data collection

Outcomes

Dual use research of concern

Policy information about dual use research of concern

Hazards

Could the accidental, deliberate or reckless misuse of agents or technologies generated in the work, or the application of information presented in the manuscript, pose a threat to:

No	Yes	
☐	☐	Public health
☐	☐	National security
☐	☐	Crops and/or livestock
☐	☐	Ecosystems
☐	☐	Any other significant area

Experiments of concern

Does the work involve any of these experiments of concern:

No	Yes	
☐	☐	Demonstrate how to render a vaccine ineffective
☐	☐	Confer resistance to therapeutically useful antibiotics or antiviral agents
☐	☐	Enhance the virulence of a pathogen or render a nonpathogen virulent
☐	☐	Increase transmissibility of a pathogen
☐	☐	Alter the host range of a pathogen
☐	☐	Enable evasion of diagnostic/detection modalities
☐	☐	Enable the weaponization of a biological agent or toxin
☐	☐	Any other potentially harmful combination of experiments and agents

Plants

Seed stocks

Novel plant genotypes

Authentication

ChIP-seq

Data deposition

☐ Confirm that both raw and final processed data have been deposited in a public database such as GEO.

☐ Confirm that you have deposited or provided access to graph files (e.g. BED files) for the called peaks.

Data access links
May remain private before publication.

Files in database submission

Genome browser session
(e.g. UCSC)

Methodology

Replicates

Sequencing depth

Antibodies

Peak calling parameters

Data quality

Software

Flow Cytometry

Plots

Confirm that:

☐ The axis labels state the marker and fluorochrome used (e.g. CD4-FITC).

☐ The axis scales are clearly visible. Include numbers along axes only for bottom left plot of group (a 'group' is an analysis of identical markers).

☐ All plots are contour plots with outliers or pseudocolor plots.

☐ A numerical value for number of cells or percentage (with statistics) is provided.

Methodology

Sample preparation

Instrument

Software

Cell population abundance

Gating strategy

☐ Tick this box to confirm that a figure exemplifying the gating strategy is provided in the Supplementary Information.

Magnetic resonance imaging

Experimental design

Design type

Design specifications

Behavioral performance measures

Imaging type(s)

Field strength

Sequence & imaging parameters

Area of acquisition

Diffusion MRI ☐ Used ☐ Not used

Preprocessing

Preprocessing software

Normalization

Normalization template

Noise and artifact removal

Volume censoring

Statistical modeling & inference

Model type and settings

Effect(s) tested

Specify type of analysis: ☐ Whole brain ☐ ROI-based ☐ Both

Statistic type for inference

(See Eklund et al. 2016)

Correction

Models & analysis

n/a | Involved in the study
- [ ] [ ] Functional and/or effective connectivity
- [ ] [ ] Graph analysis
- [ ] [ ] Multivariate modeling or predictive analysis

Functional and/or effective connectivity

Graph analysis

Multivariate modeling and predictive analysis

Addendum: Information on mouse strains used

For this study we chose mice on the C57BL/6J background.Cdh5-tdTomato/histone-GFP double reporter mice were used for the visualization of endothelial membranes and nuclei. wW crossed these mice with CX3CR1-GFP+ animals to obtain a CX3CR1-GFP x Cdh5-tdTomato/histone-GFP genotype for the visualization of endogenous fluorescence of vessel subsets and myeloid cells. Prx1-Cre R26-LSL-YFP and Prx1-Cre R26-LSL-tdRFP mice were used to visualize the mesenchymal stromal cell network.CD19-Cre R26-LSL-tdRFP mice were used to visualize B lineage lymphocytes. Fucci (fluorescent ubiquitination-based cell-cycle indicator) 474 and 639 mice were used to visualize the G1 and S/G2/M phase of the cell cycle. Adipoq-cre mTmG mice were used to label adipocytes. Mice were considered young up to an age of 25 weeks, aged mice were at least 18 months old.

This checklist template is licensed under a Creative Commons Attribution 4.0 International License, which permits use, sharing, adaptation, distribution and reproduction in any medium or format, as long as you give appropriate credit to the original author(s) and the source, provide a link to the Creative Commons license, and indicate if changes were made. The images or other third party material in this article are included in the article's Creative Commons license, unless indicated otherwise in a credit line to the material. If material is not included in the article's Creative Commons license and your intended use is not permitted by statutory regulation or exceeds the permitted use, you will need to obtain permission directly from the copyright holder. To view a copy of this license, visit http://creativecommons.org/licenses/by/4.0/
